# Angler and environmental influences on walleye *Sander vitreus* and muskellunge *Esox masquinongy* angler catch in Escanaba Lake, Wisconsin 2003–2015

**DOI:** 10.1371/journal.pone.0257882

**Published:** 2021-09-30

**Authors:** Stephanie L. Shaw, Kathryn M. Renik, Greg G. Sass

**Affiliations:** Wisconsin Department of Natural Resources, Escanaba Lake Research Station, Office of Applied Science, Madison, Wisconsin, United States of America; University of Texas Southwestern Medical Center, UNITED STATES

## Abstract

Angler trip success and catch rates are dependent upon a fishes’ vulnerability to angling. Angling vulnerability can be influenced by angler-specific attributes (i.e., bait choice, lure size, use of a guide), and individual fish traits (i.e., boldness, aggression, stress responsiveness, and memory retention). The mechanisms that function in a fishes’ angling vulnerability, and contribute to catch rate, are likely correlated with environmental factors however, the influence of environmental factors on angling vulnerability are not well understood. We used the long-term (1946 –present) compulsory creel dataset from Escanaba Lake, WI, USA to test for interactions between angling vulnerability (i.e., angler trip success and catch rates) and environmental factors to better understand these dynamics in recreational fisheries. Our objective was to test for the influence of angler associated variables and environmental factors on open water angler trip success (i.e., catch ≥ one fish) and catch rate of walleye *Sander vitreus* and muskellunge *Esox masquinongy* during 2003–2015 using a hurdle model approach. Fishing trip success and catch rates for both species were most strongly influenced by angler-related variables (i.e., guide status, bait type, the proportion of the fish population previously caught). Environmental factors associated with lower light intensity (i.e., diel period, mean daily solar radiation, solar-Julian day interaction) had a positive influence on walleye vulnerability. Lower air temperatures and lunar position (moon overhead or underfoot) and phase (gibbous’ and full moon) also had a positive effect on walleye angling. Muskellunge trip success and catch rate were positively influenced by light metrics (i.e., diel period and mean daily solar radiation) and increased with air temperature. Lunar variables (position and phase), as well as wind speed and direction also influenced muskellunge angling vulnerability. A better understanding of the influence of environmental factors on angling vulnerability is an important component of fisheries management as management goals focus on balancing fish populations and creating satisfactory catch rates to enhance the angling experience. Our results suggest that angler-specific variables, light, temperature, lunar, and weather conditions influenced species-specific angling vulnerability for walleye and muskellunge.

## Introduction

Recreational fisheries are embedded within complex social-ecological systems [1,2]. Simultaneously, fisheries are dependent on anglers and the angling sector to support conservation and management [3,4]. Accordingly, a better understanding of the influence of environmental factors on angling vulnerability is an important component of fisheries management [5–7] as management goals focus on balancing fish populations, creating satisfactory catch rates to enhance the angling experience, and providing opportunities to catch trophy-sized fish [8,9].

Angler trip success and catch rates are dependent upon a fishes’ vulnerability to angling gear [10] and often decline with increased fishing effort [11,12]. Fish angling vulnerability is described as a function of mechanisms that are influenced by the internal state of the fish, bait encounter probability, and selectivity of angling gear [10,13]. Angling vulnerability and catchability of fish is influenced by angler-specific attributes [14] such as bait choice [15,16], lure size [17–19] and use of a guide [16]. In addition, the decision for an individual fish to strike, and potentially contribute to a successful catch, is determined by a fishes’ previous experience and threat perception [12]. Boldness and aggression [20,21], stress responsiveness [22], and memory retention [10,23–25] are individual traits that influence fish angling vulnerability.

The mechanisms that function in a fishes’ angling vulnerability, that contribute to increased catch rates, are also likely correlated with environmental factors [26,27]. Piscivores, such as walleye *Sander vitreus* and muskellunge *Esox masquinongy*, exist at the apex of aquatic food webs and are believed to exhibit foraging behaviors dependent upon environmental conditions [28]. Walleye exposed to intense light conditions tend to seek physical shelter [29], but scotopic vision is an advantageous trait that contributes to increased activity in lower light and turbid conditions [30–33]. Because muskellunge are visual predators, feeding may be enhanced during specific lunar phases due to increased prey detection and vulnerability associated with greater lunar illuminance [34,35]. Anglers are cognizant of potential environmental effects on fish behavior and use this information to increase fishing success, often exploiting “Solunar” tables that forecast fish feeding times with day and hour predictions correlated with sun and lunar observations [36,37]. Nevertheless, many environmental factors may influence fish angling vulnerability, and outside of a few angler-specific factors, light, lunar, and seasonal attributes, most factors are not well understood or studied [16,36].

Limited information is available on the potential influences of environmental factors on freshwater angler catch rates and fishing trip success, as most studies are primarily marine-oriented and focused on lunar effects. Further, a pertinent question regarding which factors (e.g., angler experience, weather) influenced the ability of anglers to catch fish was identified in a recent study that surveyed recreational fisheries experts and multiple sectors, such as industry and government, as an important topic of prospective research [4]. Therefore, we tested for the interactions between angler trip success and catch rates, fish vulnerability to angling, and environmental variables to better understand these dynamics in recreational fisheries. The objective of our study was to quantify the influence of multiple angler associated and environmental factors on angler trip success and catch rates of walleye and muskellunge using information from the long-term compulsory creel dataset available at Escanaba Lake, Wisconsin during 2003–2015. The compulsory creel census data available from Escanaba Lake (1946 –present) provides an unprecedented opportunity to test for interactions among anglers, fish, and environmental factors due to its long-term duration on a relatively large, natural lake.

## Methods

### Study site

**Fig 1:**
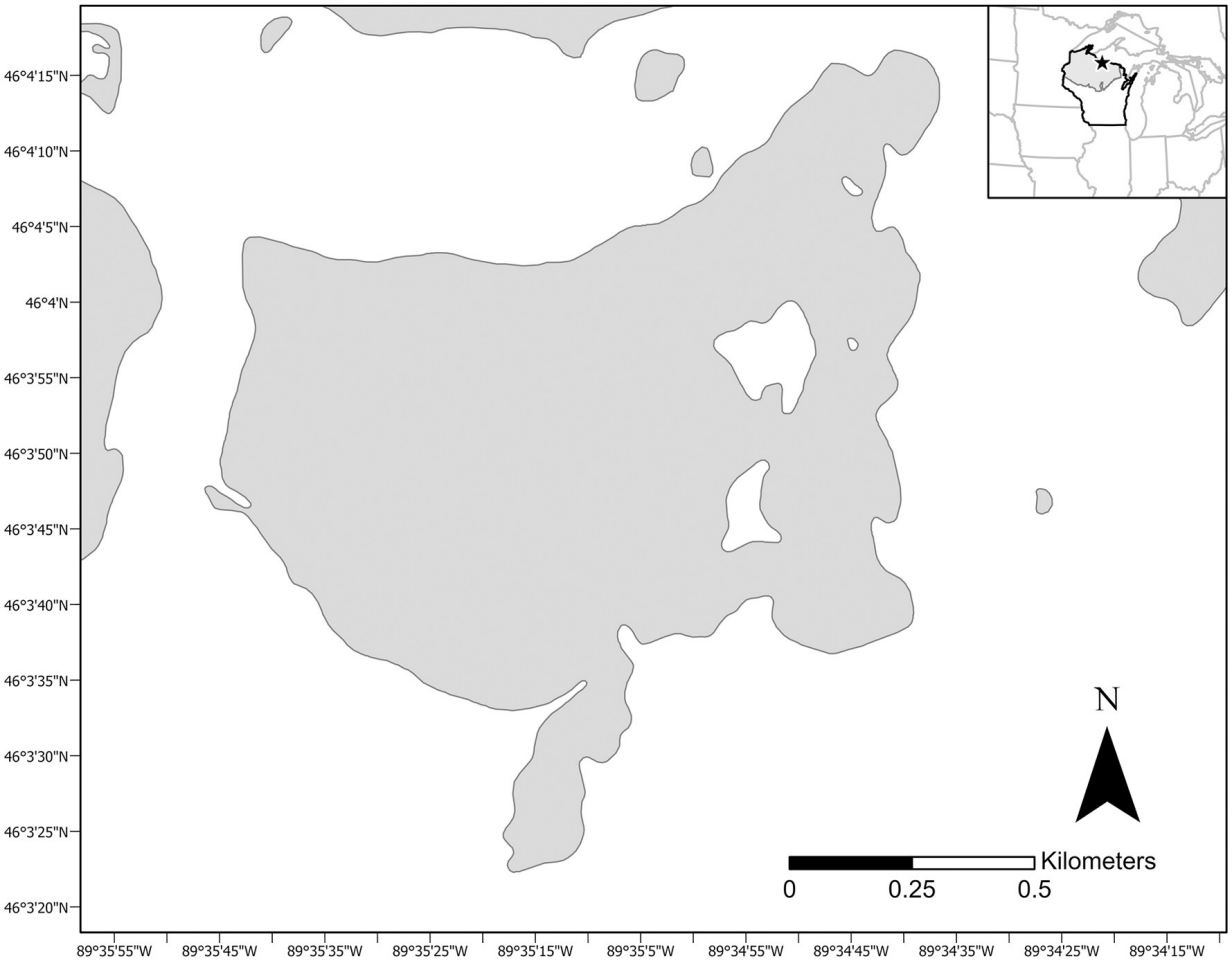
The study site, Escanaba Lake, WI, USA. Escanaba Lake is a 119-ha designated research lake located in the Northern Highland American Legion State Forest of Vilas County, WI (46°03′49.2″ N, 89°35′11.7″ W).

Escanaba Lake is a mesotrophic, drainage lake located in the Northern Highland American Legion State Forest of Vilas County, WI (46°03′49.2″ N, 89°35′11.7″ W; Fig 1, ArcPro 2.6). The 119-ha lake has a shoreline length of 8.2 km and mean depth of 4.3 m [38]. The shoreline of Escanaba Lake is undeveloped, with the exception of a research facility and one public boat launch. At least 24 fish species inhabit Escanaba Lake [38–41]; however, the most targeted species of the sport fishery include walleye, muskellunge, yellow perch *Perca flavescens*, smallmouth bass *Micropterus dolomieu*, and northern pike *Esox lucius* [42].

In 1946, Escanaba Lake was designated a research lake preceding establishment of the Wisconsin Department of Natural Resources, Northern Highland Fishery Research Area [(NHFRA); 16]. The Escanaba Lake sport fishery is currently unregulated by length, bag, or seasonal restrictions on all fish species except walleye, which are currently regulated by a 711-mm minimum length limit and daily bag limit of one fish [16,43]. A compulsory creel census is continuously conducted on Escanaba Lake and daily cost-free permits are issued by a creel clerk upon mandatory angler check-in at the research station. Anglers are required to return permits at the conclusion of their fishing trip and participate in an interview that acquires information regarding angler demographics, species-specific directed effort, bait type used (live or artificial), guided status (guided or not), and number of fish caught and released or harvested [16]. The annual fishing season for Escanaba Lake begins at the start of the open water season (i.e., ice-off in a given year) and closes with the end of the ice-on season the following year. Thus, the annual season for Escanaba Lake encompasses the open water and ice-covered season for a given yearly transition.

We used the Escanaba Lake long-term compulsory creel dataset to test for environmental influences on walleye and muskellunge angler catch rates during 2003–2015. We subset the data to represent only the open-water period—generally coinciding with late April to late October—due to greater angler effort and the differences in angling gear and strategy used for open water versus ice fishing [16]. Angler data was summarized by trip (i.e., all anglers in a single boat). Catch per unit effort CPUE included the total number of walleye or muskellunge caught by all anglers per trip divided by effort which was calculated as the sum of total angler hour(s) per fishing trip. Previous research found that trip specific variables associated with angler choice, such as bait type (live or artificial) and guide status (guided or not), influenced the probability of fishing trip success and catch rate of walleye [16]. Thus, these variables were also included in our analyses for each species to test for differences in fishing trip success and CPUE that is related to trip-specific angler choice variables. Additional metrics associated with angler activity included the daily density of trips on Escanaba Lake (i.e., daily total trips/ha). Angler density was assumed to be positively correlated with environmental variables that may influence CPUE and trip success. We hypothesized that trip success and CPUE of walleye and muskellunge would increase with angler density.

Angling vulnerability has been shown to decrease for some species in relation to increased exposure to angling tactics over time suggesting learning behavior and lure avoidance [27,44–48]. We hypothesized that similar effects would influence walleye and muskellunge; as a larger proportion of the population is caught and released over the annual season, catch rates and angler trip success may decline due to learned avoidance behaviors. For example, Wegener et al. (2018) found that largemouth bass *Micropterus salmoides* catch rates declined within a season under consistent angling effort, whereas a two-month fishery closure treatment resulted in elevated catch rates after fishery closure and then a decline [49]. Thus, we included a variable to test for the potential effects of angling exposure and learning which was the proportion of the walleye and muskellunge population that had been caught and released to a given date in the fishing season (i.e., the cumulative catch of walleye or muskellunge/species abundance). Walleye and muskellunge abundance were estimated annually during spring on Escanaba Lake using standardized mark-recapture methods [43,50]. Finally, trip success and angler CPUE were assumed to relate to population density. We hypothesized that trip success and CPUE would be positively related to walleye or muskellunge annual density (walleye or muskellunge/ha).

### Environmental factors

Multiple environmental factors have been associated with changes in fish behavior and angling vulnerability. Behaviors associated with foraging activity, spawning, aggression/territoriality, or movement patterns (e.g., seasonal or daily home range) can influence encounter probability with anglers and angling vulnerability (i.e., potential to chase or strike a lure or live bait). Seasonal progression of temperature and light triggers spawning activity in the spring and heightens foraging activity in the fall for walleye and muskellunge [16,51]. Higher temperatures during the summer also increase metabolism and may ultimately influence activity levels seasonally or daily [52]. We hypothesized that factors such as mean hourly air temperature (°C), and mean daily solar radiation (W/m^2^) would influence angling vulnerability by influencing movement range and foraging patterns and ultimately, encounter probability with anglers, as well as the probability that a fish would strike a lure or live bait. Mean daily solar radiation was not calculated on a 24-hour time scale, but only included open fishing hours. Solar radiation is influenced by solar altitude as well as cloud cover. Mean solar radiation was moderately correlated with day of the year (Julian day; r = -0.46), but not strongly with solar elevation (degrees above the horizon; r = 0.07). Thus, mean daily solar radiation was assumed to represent cloud cover as well as seasonal or diel differences in solar intensity due to annual changes in sun position.

Daily environmental factors, such as light availability (i.e., diel periods or cloud cover) and storm front activity, have also been associated with increased fish activity [20,24,38]. We hypothesized that walleye angling vulnerability would increase during periods of lower light intensity due to their low light adaptations and increased foraging activity, while muskellunge angling vulnerability may be associated with periods of higher light intensity due to increased prey activity and observed response in movement [26,27,29,53,54]. The factor diel period was used to represent daily cycles of light intensity, while mean daily solar radiation was assumed to represent daily changes in cloud cover and light intensity due to sun elevation as previously described. The diel period for each angling trip was categorized by time of day (dawn, day, or dusk). The NHFRA designates fishing hours and there is no legal fishing allowed at night; each day begins at 0400 h and concludes at dark, with transitional closing hours to reflect seasonal changes in daylight. Trips that occurred during spring (March and April) and fall (October and November) before 0900 h were classified as ‘dawn’, between 0900 and 1800 h as ‘day’, and after 1800 h as ‘dusk’. For angling that occurred during summer months (May–September), fishing trips that occurred before 0800 h were classified as ‘dawn’, between 0800 and 1900 h as ‘day’, and after 1900 h as ‘dusk’.

Storm front activity is often anecdotally associated with changes in angler catch rates. We hypothesized that storm activity would influence angling vulnerability for walleye and muskellunge by influencing foraging patterns; however, no published information to our knowledge is available to support this hypothesis. The variables of daily trend in barometric pressure (millibars; mb), total hourly precipitation (cm), mean hourly wind speed (m·s^-1^), and daily resultant wind direction (°360) were used to indicate daily weather patterns associated with front activity. Barometric pressure is an indicator of atmospheric stability or passing fronts. Barometric pressure (mb) was recorded twice daily (0800 and 1800 h; Central Standard Time [CST]) at the NHFRA Escanaba Lake Research Station and was used to calculate the daily change in barometric pressure (mb) for each fishing day (magnitude of change and direction of trend; positive or negative). Meteorological observations during 2003–2015, except barometric pressure trend, were obtained from the North Temperate Lakes Long-Term Ecological Research meteorological dataset field site location at Noble F. Lee Municipal airport in Woodruff, WI, which is about 18 km from Escanaba Lake. Total hourly precipitation (cm) was summed for 5-minute intervals during periods of detectable precipitation. Average hourly wind speed and daily resultant wind direction were measured at 3 m above ground and averaged from 1-minute samples. Wind direction values were obtained by converting the 1-minute wind speeds and directions for each hour into a single daily vector. Resultant wind direction is the direction of this vector and was measured to the nearest degree based on a 360° compass (360° being from the North, 180° being from the South, 0° representing calm winds).

Lunar phases have been associated with the activity patterns of marine and freshwater aquatic species and lunar phase and position have been identified as factors influencing catch rates of muskellunge by anglers and scientists [36,55,56]. Thus, we hypothesized that lunar phase and lunar position would influence angling vulnerability. The lunar phase that coincided with each angling trip was obtained via the ‘lunar.phase’ function in R package ‘lunar’ [57] and a shift of– 5 from coordinated universal time was applied to represent the appropriate time zone for Escanaba Lake (CST). We further obtained the time when the moon position was ‘overhead’ (or meridian passing) using www.timeanddate.com for Boulder Junction, Vilas County, WI that aligned for each day an angling trip occurred. This also allowed the moon position ‘underfoot’ (i.e., when the moon is on the opposite side of the earth from the study location) to be determined for each fishing day by the addition of twelve hours to the moon overhead time. If the ‘overhead’ or ‘underfoot’ designated time occurred within the time frame of an angling trip, it was specified as such; otherwise, it was denoted as ‘neither’.

Additional variables that could be assumed to influence fish angling vulnerability (e.g., Julian day, season, water temperature, and solunar value) were considered, but were found to be highly correlated with other variables (e.g., air temperature, solar radiation, lunar phase, and lunar position). Thus, to avoid redundancy, we chose those variables that we thought categorized the important environmental factors in sufficient detail and with as few variables as possible.

### Statistical analyses

First, we tested for temporal trends, during 2003–2015, for each continuous variable using simple linear regression (i.e., population density, annual CPUE, the sum total of angler effort per year, mean effort per trip, total precipitation per year, and mean monthly precipitation) or a general linear model with month and year as variables (i.e., mean monthly air temperature, mean monthly solar radiation, and mean monthly wind speed). Linear models were run using the lm and glm functions as part of the stats package in Program R [58]. Weather related trends were evaluated using the broader dataset of daily observations available from the North Temperate Lakes Long-Term Ecological Research meteorological dataset field site location at Noble F. Lee Municipal airport in Woodruff, WI (i.e., not only those weather observations associated with angling trips but all daily observations). Non-normally distributed variables were natural log transformed to meet model assumptions of normality.

Then, all continuous independent variables were centered (mean = 0) and scaled (SD = 1) prior to model fitting. Data was centered to a mean of zero by subtracting the variable mean from each variable value and then scaled by dividing the variable values by the centered variable SD [scale function Program R; 58]. We tested for correlation among scaled and centered independent variables using Pearson correlation coefficient [chart.Correlation function Program R; 58, 59]. Variable correlation ≥ 0.8 were considered strongly correlated, and in the case of strong correlations one of the variable pair would be removed from analysis. We used hurdle models to test for environmental factors influencing angler trip success and CPUE of walleye and muskellunge. A hurdle model is a two-part model that is directly suited to handle zero-inflated datasets [60]. The first part of the model addressed zero-inflation by evaluating fishing trip success in a binomial format (i.e., catch of at least one target fish indicated a successful trip or no catch which indicated and unsuccessful trip). The second component is a positive truncated model that evaluated CPUE from only successful trips. Catch rate (CPUE) was natural log transformed and positive truncated models were fit assuming a normal distribution [family Gaussian, glmmTMB function; glmmTMB package; 58, 60]. We used the ‘glmmTMB’ R package [58,60] to fit the hurdle models for each species where ’year’ was treated as a random effect and all other variables were fixed effects. The full model for each species included the complete set of candidate variables (i.e., guide + effort + bait + daily trip density + proportion of the population caught and released + population density + barometric pressure trend + total precipitation + mean wind speed + resultant wind direction + wind speed*wind direction + mean solar radiation +lunar phase + lunar position + diel period + mean hourly air temperature). We used Akaike’s information criterion (AIC_c_) model selection procedures to determine the top model for walleye and muskellunge. Due to the high number of potential model combinations, we did not conduct an exhaustive selection. Rather, we fit a series of candidate models following general hypotheses surrounding variable importance including: a drop-one method where each variable was dropped one at time, a drop variable group method where similar categories of variables were removed one at a time coinciding with hypotheses regarding variable types discussed previously in the Methods (i.e., angler choice metrics, all angler metrics, fish population metrics, lunar metrics, light intensity metrics, storm metrics, seasonal metrics), and finally a forward selection process where each variable was successively removed based on least significance in the model summary output until the null model was reached. Models with delta AIC_c_ (ΔAIC_c_) values ≥ 8 were considered to have little support, while those that had ΔAIC_c_ < 3 were considered plausible [61,62]. Models that differed by ΔAIC_c_ values < 2.0 were considered to be similarly likely [63]. A pseudo adjusted R^2^ value based on the likelihood ratio of a given model to the null model (random effect term only) was calculated using the r.squaredLR function from the MuMIn package in Program R [58,64,65]. A variable importance metric was calculated for each variable by summing the model weight (*w*_*t*_) for each model in which that variable was included [63]. The top model for each species and analysis (i.e., trip success and CPUE) were evaluated for significant outliers and model deviations using QQ-plots and model residual versus predicted values [simulateResiduals and plotDHARMa functions from the DHARMa package in Program R; 58, 66]. Variable effect sizes Cohen’s d were calculated from the full model using the z_to_d function from the effectsize package in Program R [67].

## Results

**Fig 2:**
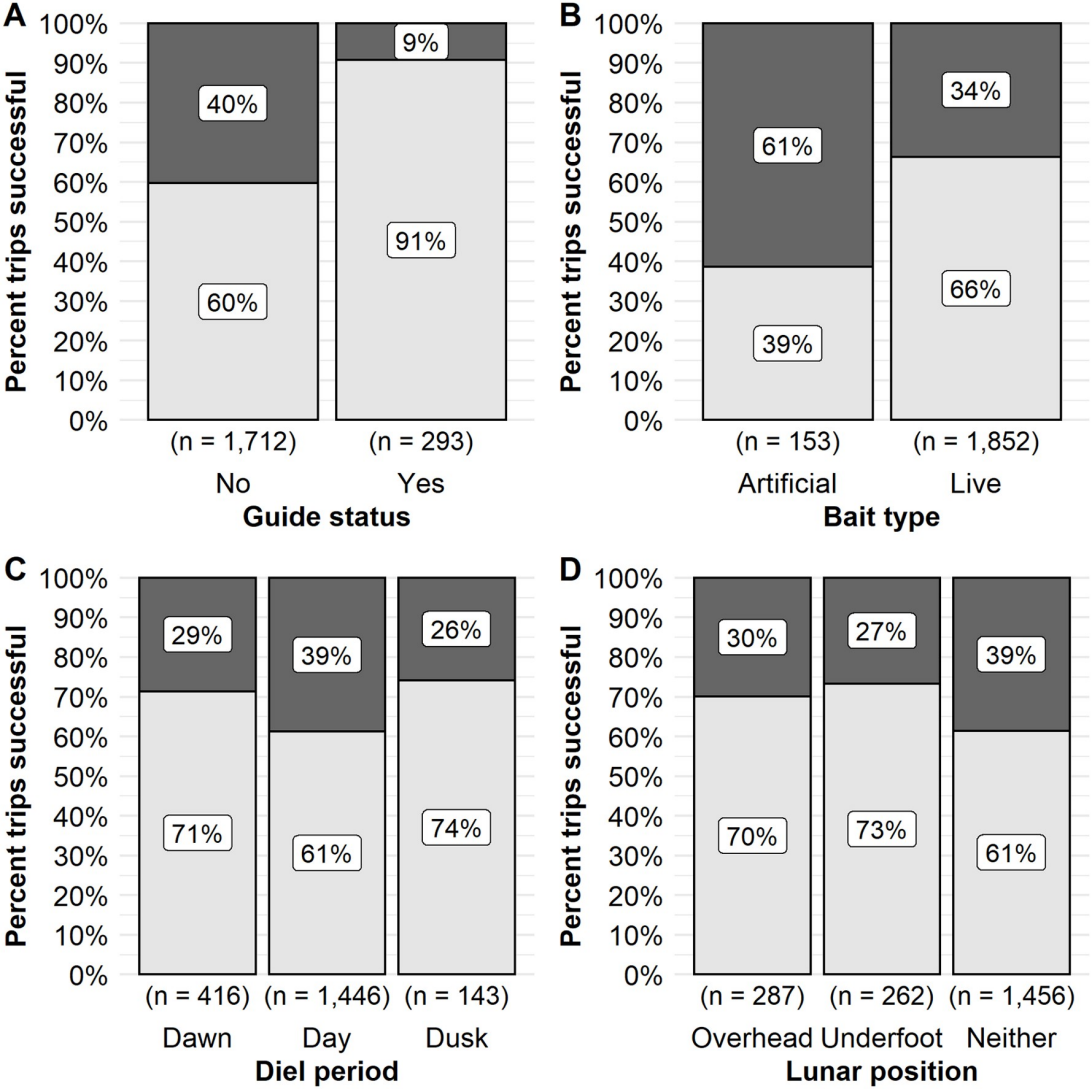
The observed proportion of trips successful for walleye. The observed proportion of trips successful for model variables guide status (A), bait type used (B), diel period (C) and lunar position (D) on Escanaba Lake, WI, USA. The observed proportion of trips successful are indicated by the lower, light grey, portion of the column. Unsuccessful trips are indicated by the dark grey, upper portion of the column. The percent successful or unsuccessful is indicated by the label within each section. The sample size of trips included in each category is indicated below the bar.

**Fig 3:**
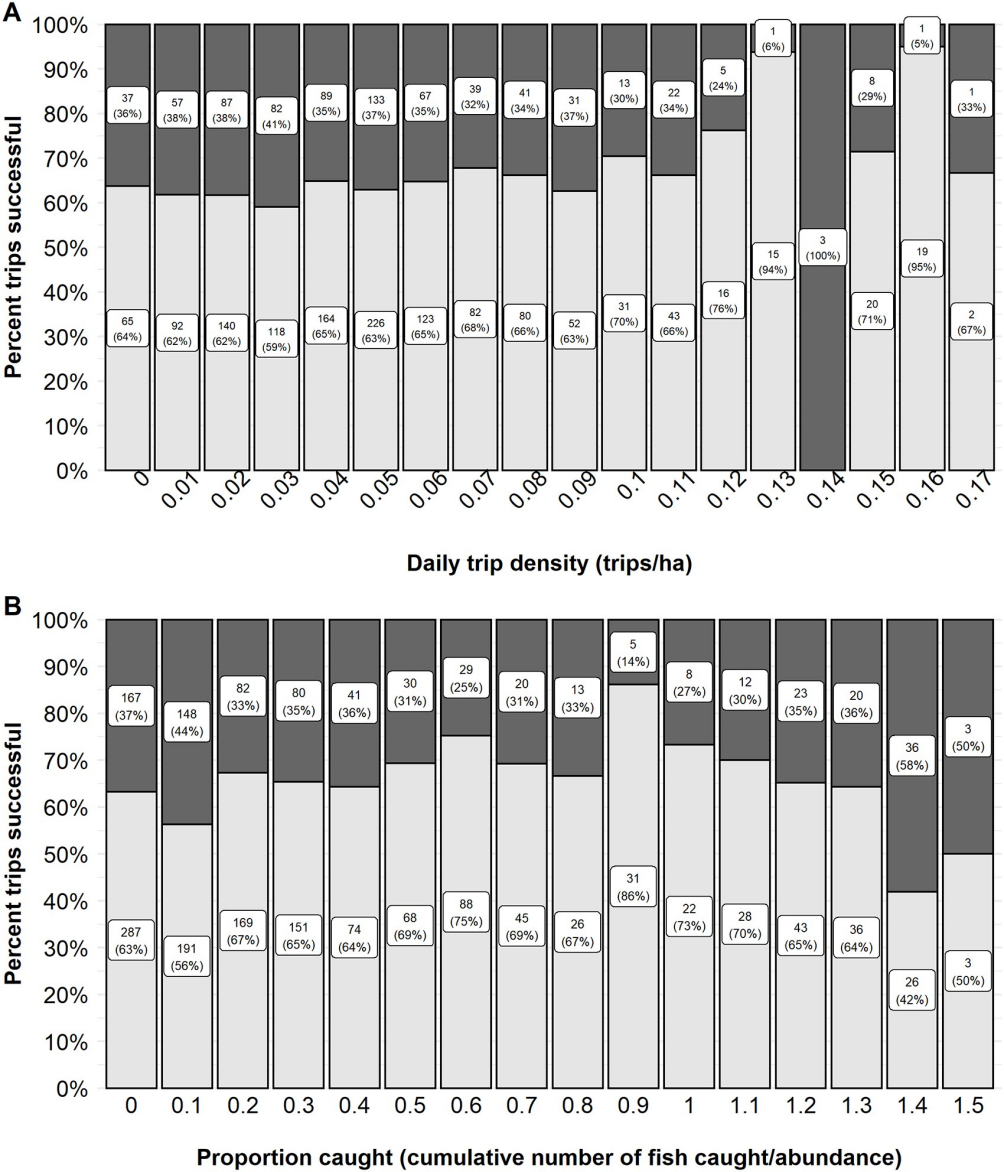
The observed proportion of trips successful for walleye. The observed proportion of trips successful for model variables daily trip density (A) and proportion caught (B) on Escanaba Lake, WI, USA. Continuous variables were binned to present observed proportion successful. The observed proportion of trips successful are indicated by the lower, light grey, portion of the column. Unsuccessful trips are indicated by the dark grey, upper portion of the column. The label within each section indicates the sample size (top number) and the observed percent successful or unsuccessful (bottom number in parentheses).

**Fig 4:**
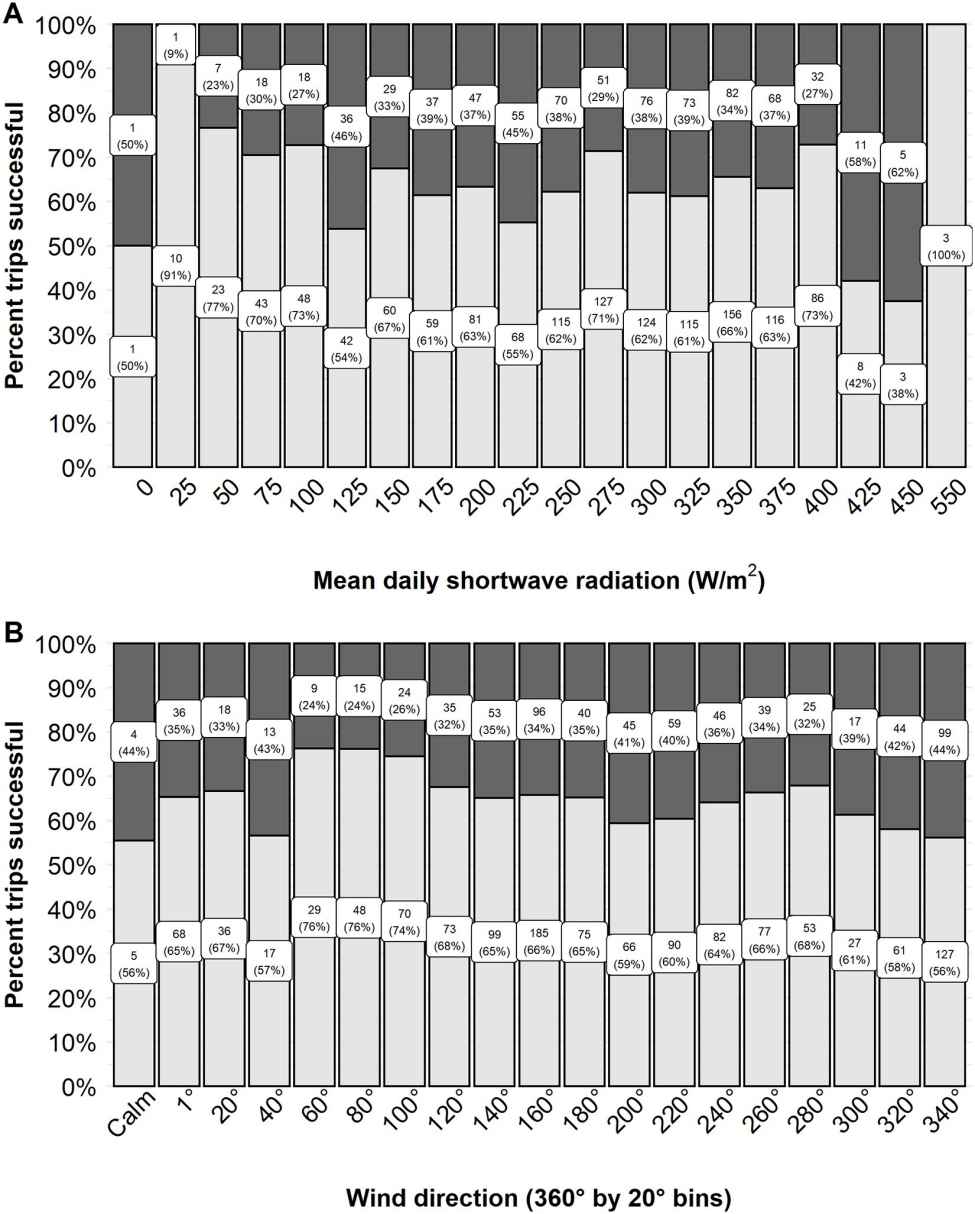
The observed proportion of trips successful for walleye. The observed proportion of trips successful for model variables mean daily solar radiation (W/m^2^; A) and daily peak wind direction (°360; B) on Escanaba Lake, WI, USA. Continuous variables were binned to present observed proportion successful. The observed proportion of trips successful are indicated by the lower, light grey, portion of the column. Unsuccessful trips are indicated by the dark grey, upper portion of the column. The label within each section indicates the sample size (top number) and the observed percent successful or unsuccessful (bottom number in parentheses).

Adult walleye and adult muskellunge density did not differ significantly over the duration of the study, 2003–2015 (Fig 1 in S1 Appendix). Total annual angler effort did not significantly differ over time for walleye (Fig 2 in S1 Appendix), but significantly declined for muskellunge (F_1,11_ = 8.5, p-value = 0.01; Fig 3 in S1 Appendix). Mean effort per trip (natural log(mean hours per trip)) significantly increased for walleye and muskellunge from 2003–2015 (walleye F_1,2003_ = 6.5, p-value = 0.01; muskellunge F_1,2769_ = 8.0, p-value = 0.005; Figs 2B and 3B in S1 Appendix). Adult walleye CPUE did not significantly differ over time. However, adult muskellunge CPUE significantly declined during 2003–2015 (F_1,2769_ = 15.7, p-value < 0.001; Fig 4B in S1 Appendix). For muskellunge, CPUE for positive catch trips did not significantly differ over time, but rather there was a decline in trip success observed, with a mean percent trips successful of 21% (± 6% SD) during 2003–2008 to a mean 15% (± 2% SD) of trips successful during 2009–2015.

**Fig 5:**
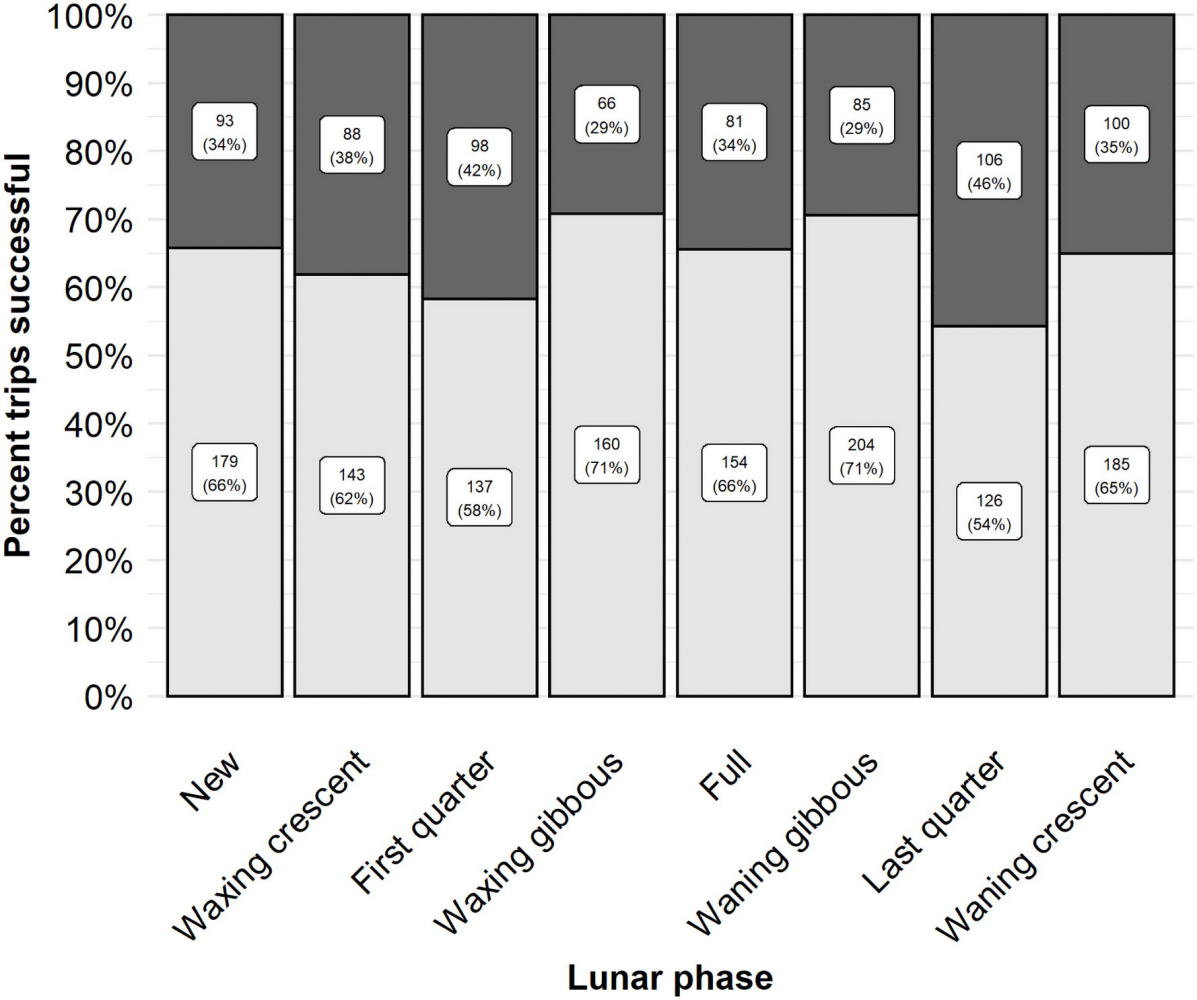
The observed proportion of trips successful for walleye. The observed proportion of trips successful for model variable lunar phase on Escanaba Lake, WI, USA. Continuous variables were binned to present observed proportion successful. The observed proportion of trips successful are indicated by the lower, light grey, portion of the column. Unsuccessful trips are indicated by the dark grey, upper portion of the column. The label within each section indicates the sample size (top number) and the observed percent successful or unsuccessful (bottom number in parentheses).

**Fig 6:**
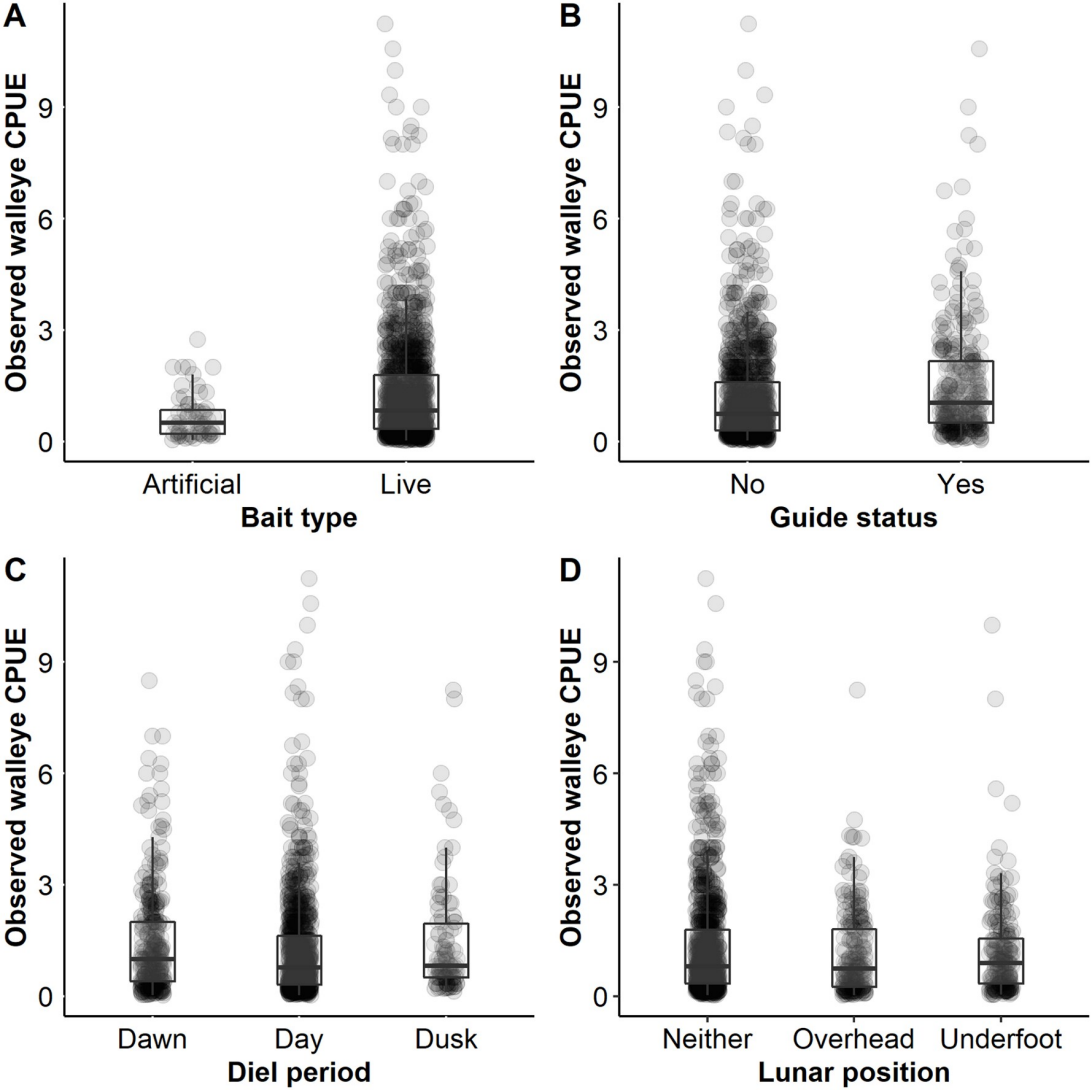
Observed walleye catch rate. Observed walleye *Sander vitreus* catch per unit effort (CPUE) for targeted walleye only angling trips on Escanaba Lake, Vilas County, WI during 2003–2015. Box plots represent the median catch rate (middle bar) and upper and lower quartiles (boundaries of the box) in relation to bait type (A), guide status (B), diel period (C), and lunar position (D). Observed values are represented by light grey points.

**Fig 7:**
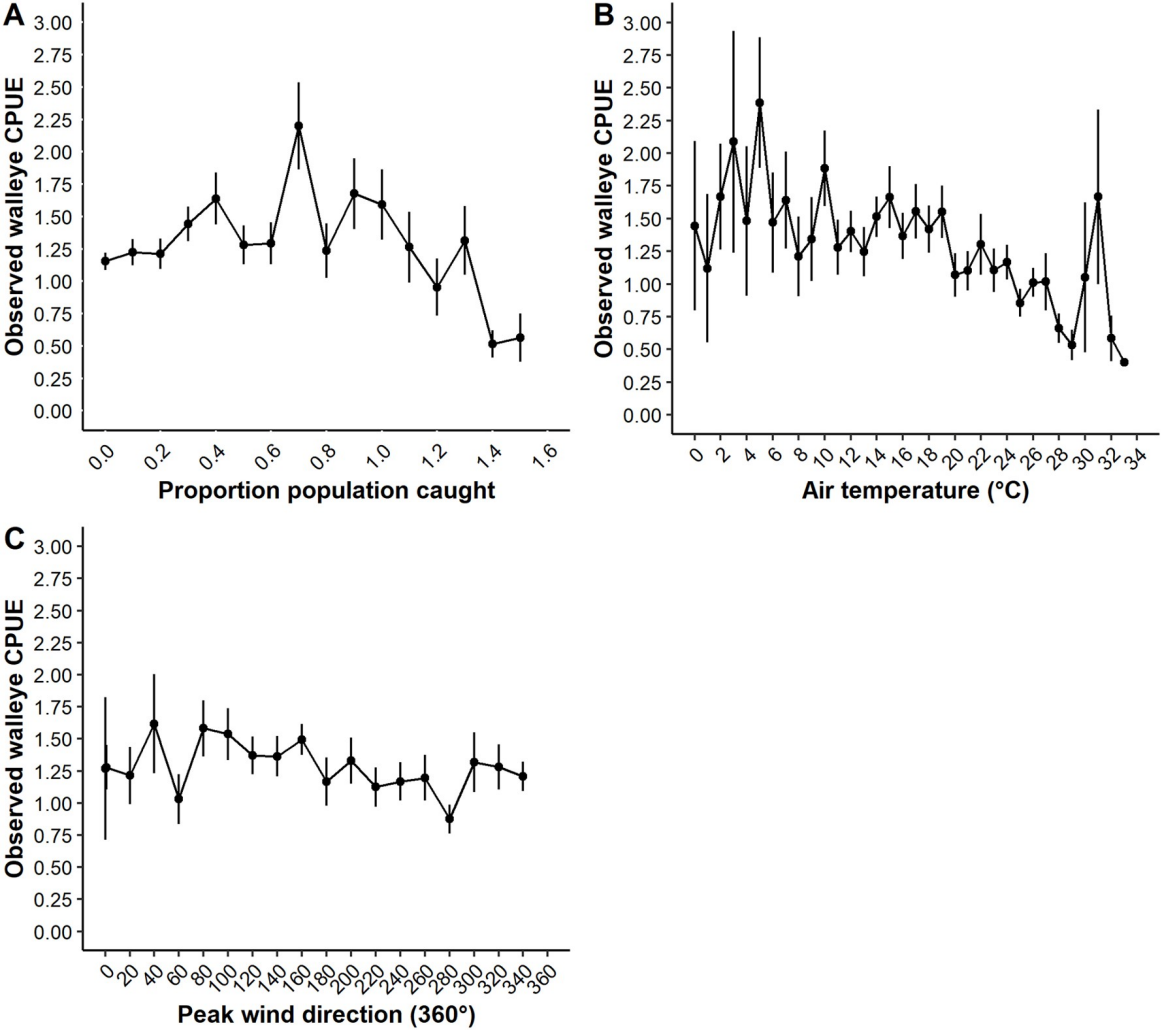
Observed walleye catch rate. Observed walleye *Sander vitreus* mean catch per unit effort (CPUE) for targeted walleye only angling trips on Escanaba Lake, Vilas County, WI during 2003–2015, in relation to the proportion of the walleye population caught (A), air temperature (°C; B), and daily peak wind direction (°360; C). Observed values are represented by the mean catch rate and error bars are ± SE.

**Fig 8:**
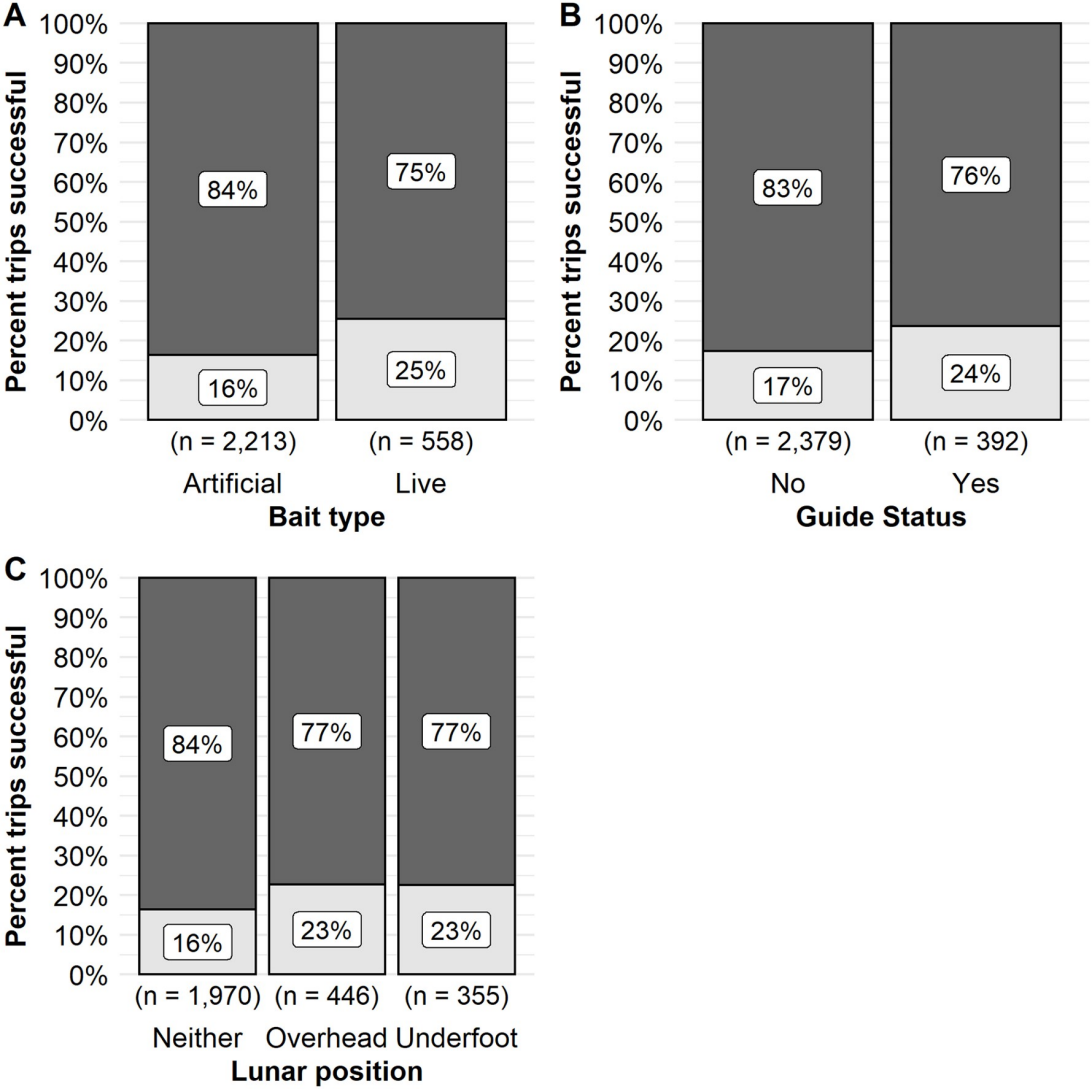
The observed proportion of trips successful for muskellunge. The observed proportion of successful trips for model variables bait type (A), guide status (B), lunar position (C) on Escanaba Lake, WI, USA. The observed proportion of trips successful are indicated by the lower, light grey, portion of the column. Unsuccessful trips are indicated by the dark grey, upper portion of the column. The percent successful or unsuccessful is indicated by the label within each section. The sample size of trips included in each category is indicated below the bar.

Total annual precipitation and mean daily precipitation did not significantly differ during 2003–2015 (Fig 5 in S1 Appendix). Mean monthly air temperature, mean monthly solar radiation, and mean monthly wind speed did not significantly differ among years during 2003–2015 (Figs 6–8 in S1 Appendix). There were no strong correlations (r ≥ 0.8) observed between scaled and centered variables, thus all variables described were included in the full models.

### Walleye

#### Trip success

There was a total of 2005 trips during 2003–2015 that targeted only walleye on Escanaba Lake, WI, USA. The probability of a successful walleye trip (i.e., catching at least one walleye) during 2003–2015 was 0.64 (95% CI 0.62–0.66) and ranged from a low of 0.46 in 2004 to a high of 0.73 in 2007. There were several models that were considered plausible (ΔAIC_c_ ≤ 8.0) and the top four models did not differ appreciably from each other (ΔAIC_c_ ≤ 2.0; Table 1 in S2 Appendix and Fig 1 in S2 Appendix). Variable importance weighting suggested that all angler metrics (i.e., guide status, bait type, and trip density) were of high relative importance (i.e., included in all weighted models and having variable weights = 1.0; Table 1). Guide status and bait type had the highest effect size relative to all other parameters (Table 1). The effect sizes were considered moderate (guide status) to small (bait type) based on Cohen’s interpretation [67]. Additional variables with high relative importance (variable importance weight = 1.0) included the proportion of the walleye population captured, wind direction, solar radiation, diel period and lunar position. Lunar phase had a similar importance at 0.99 (Table 1). However, all of these variables had very small effect sizes (< 0.20; Table 1) [67].

**Table 1:** Walleye *Sander vitreus* full hurdle model parameter results for trip success and positive truncated catch rate data (CPUE) of targeted walleye trips only from Escanaba Lake, WI during 2003–2015.

*Walleye trip success*								
Variable	Variable type	Coefficient (± SE)	Lower 95% CI	Upper 95% CI	z - value	p - value	Cohen’s d	Variable importance weight
Guide [Yes]	angler choice	1.86 (± 0.22)	1.43	2.28	8.60	0.000	0.38	1.00
Bait [Live]	angler choice	0.96 (± 0.18)	0.60	1.32	5.24	0.000	0.23	1.00
Trip density	angler	0.24 (± 0.06)	0.13	0.35	4.27	0.000	0.19	1.00
Proportion caught	population	-0.29 (± 0.11)	-0.50	-0.07	-2.63	0.009	-0.12	1.00
Walleye density	population	0.03 (± 0.14)	-0.25	0.30	0.18	0.855	0.01	0.06
Barometric pressure	storm	0.09 (± 0.06)	-0.02	0.20	1.55	0.122	0.07	0.69
Precipitation	storm	0.02 (± 0.04)	-0.07	0.11	0.43	0.669	0.02	0.06
Wind speed	storm	0.04 (± 0.06)	-0.08	0.17	0.68	0.497	0.03	0.30
Wind direction	storm	-0.14 (± 0.05)	-0.24	-0.03	-2.55	0.011	-0.11	1.00
Wind interaction	storm	0.04 (± 0.06)	-0.08	0.15	0.64	0.525	0.03	0.12
Air temperature	seasonal	-0.01 (± 0.07)	-0.14	0.13	-0.10	0.920	0.00	0.06
Solar radiation	seasonal/light	-0.13 (± 0.06)	-0.25	-0.01	-2.05	0.041	-0.09	1.00
Diel [Day]	light	-0.41 (± 0.15)	-0.70	-0.11	-2.72	0.006	-0.12	1.00
Diel [Dusk]	light	0.55 (± 0.24)	0.08	1.02	2.29	0.022	0.10	1.00
Lunar phase [Full]	lunar	0.12 (± 0.21)	-0.30	0.54	0.56	0.578	0.02	0.99
Lunar phase [Last quarter]	lunar	-0.27 (± 0.21)	-0.67	0.14	-1.29	0.198	-0.06	0.99
Lunar phase [New]	lunar	0.2 (± 0.20)	-0.19	0.60	1.01	0.314	0.04	0.99
Lunar phase [Waning crescent]	lunar	0.2 (± 0.20)	-0.19	0.59	0.99	0.322	0.04	0.99
Lunar phase [Waning gibbous]	lunar	0.47 (± 0.20)	0.07	0.86	2.29	0.022	0.10	0.99
Lunar phase [Waxing crescent]	lunar	-0.003 (± 0.21)	-0.41	0.40	-0.01	0.988	0.00	0.99
Lunar phase [Waxing gibbous]	lunar	0.52 (± 0.22)	0.10	0.94	2.41	0.016	0.11	0.99
Lunar position [Overhead]	lunar	0.3 (± 0.16)	-0.02	0.61	1.85	0.064	0.08	1.00
Lunar position [Underfoot]	lunar	0.55 (± 0.17)	0.22	0.88	3.23	0.001	0.14	1.00
*Walleye CPUE*								
Variable	Variable type	Coefficient (± SE)	Lower 95% CI	Upper 95% CI	z - value	p - value	Cohen’s d	Variable importance weight
Guide [Yes]	angler choice	0.07 (± 0.01)	0.04	0.09	4.56	5.04E-06	0.254	1.00
Bait [Live]	angler choice	0.10 (± 0.03)	0.05	0.16	3.86	1.14E-04	0.215	1.00
Trip density	angler	0.01 (± 0.01)	0.00	0.02	1.86	0.063	0.104	0.12
Proportion caught	population	-0.04 (± 0.01)	-0.06	-0.02	-3.65	0.000	-0.203	1.00
Walleye density	population	0.001 (± 0.02)	-0.03	0.04	0.08	0.934	0.005	0.03
Barometric pressure	storm	0.001 (± 0.01)	-0.01	0.01	0.12	0.907	0.006	0.02
Precipitation	storm	0.004 (± 0.004)	0.00	0.01	0.89	0.376	0.049	0.04
Wind speed	storm	-0.002 (± 0.01)	-0.01	0.01	-0.26	0.794	-0.015	0.03
Wind direction	storm	-0.01 (± 0.01)	-0.02	0.00	-1.49	0.137	-0.083	0.95
Wind interaction	storm	-0.003 (± 0.01)	-0.02	0.01	-0.49	0.623	-0.027	0.03
Air temperature	seasonal	-0.02 (± 0.01)	-0.04	-0.01	-2.67	0.008	-0.149	1.00
Solar radiation	seasonal/light	-0.01 (± 0.01)	-0.02	0.00	-1.30	0.192	-0.073	0.08
Diel [Day]	light	-0.01 (± 0.01)	-0.04	0.02	-0.47	0.640	-0.026	0.86
Diel [Dusk]	light	0.06 (± 0.02)	0.01	0.10	2.31	0.021	0.129	0.86
Lunar phase [Full]	lunar	-0.01 (± 0.02)	-0.05	0.04	-0.19	0.850	-0.011	0.12
Lunar phase [Last quarter]	lunar	-0.04 (± 0.03)	-0.09	0.01	-1.58	0.114	-0.088	0.12
Lunar phase [New]	lunar	-0.01 (± 0.02)	-0.06	0.03	-0.49	0.627	-0.027	0.12
Lunar phase [Waning crescent]	lunar	-0.04 (± 0.02)	-0.08	0.01	-1.62	0.104	-0.091	0.12
Lunar phase [Waning gibbous]	lunar	-0.001 (± 0.02)	-0.05	0.04	-0.06	0.953	-0.003	0.12
Lunar phase [Waxing crescent]	lunar	-0.02 (± 0.02)	-0.07	0.02	-0.95	0.343	-0.053	0.12
Lunar phase [Waxing gibbous]	lunar	-0.02 (± 0.02)	-0.07	0.03	-0.89	0.371	-0.050	0.12
Lunar position [Overhead]	lunar	-0.04 (± 0.02)	-0.07	0.00	-2.21	0.027	-0.123	0.99
Lunar position [Underfoot]	lunar	-0.02 (± 0.02)	-0.06	0.01	-1.38	0.167	-0.077	0.99

Walleye *Sander vitreus* full hurdle model parameter estimates ± SE and 95% confidence intervals, z-value, p-value, effect size (Cohen’s d) with 95% confidence intervals, and variable importance weighting derived from AIC_c_ model selection procedures for trip success and positive truncated catch rate data (CPUE), for targeted walleye trips only from Escanaba Lake, WI during 2003–2015. Variable type indicates the categorical designation for each variable used in model selection.

Angler specific variables that influenced walleye trip success included the use of a guide, the use of live bait, and the density of trips on the lake on a given day. Anglers that used a guide had a higher predicted odds of success (0.77, 95% CI 0.62–0.87) relative that those that did not use a guide (0.34 odds of success (95% CI 0.23–0.47). Observed unguided trips on Escanaba Lake were only 60% successful relative to guided trips which were 91% successful during the time period (Fig 2A). Anglers that used live bait when fishing for walleye had a predicted odds of success of 0.58 (95% CI 0.47–0.68) as opposed to those that used artificial bait only, which had a predicted probability of success of 0.34 (95% CI 0.23–0.47). Observed live bait trips were 66% successful during the time period whereas artificial only trips were 39% successful (Fig 2B). There was a positive relationship between trip density and walleye angler trip success (Table 1). The odds of a successful walleye trip ranged from 0.26 (95% CI 0.17–0.39) at a density of 0.008 trip/ha (i.e., the only trip on the lake, minimum observed) to a predicted odds of success of 0.57 (95% CI 0.40–0.73) at a density of 0.18 trip/ha (i.e., 21 total trips on the lake, maximum observed; Table 1 and Fig 3A). Observed trip success showed relatively similar success rates at low to moderate trip densities (i.e., 1 trip to 11 trips) ranging from 59% to 63% successful with increases observed in success rates at 12 trips and above (≥ 0.1 trip/ha), range 66% to 100% (Fig 3A). However, sample size at higher trip densities was limited relative to low or moderate densities. We hypothesized that walleye may experience learning and lure avoidance as the season progressed and more and more of the individuals in the population were angled. The proportion of the walleye population that was caught was predicted to have a negative effect on trip success (Table 1). The predicted odds of a successful trip were 0.49 (95% CI 0.34–0.65) at the start of the season when few to no walleye had yet been angled. The odds of success declined to 0.17 (95% CI 0.08–0.32) when the proportion captured reached 150% which would suggest that a high proportion of the population had been angled at least once and some fish multiple times that year. The effect size was considered very small (Table 1). The observed trip success was similar across the range of the proportion of the population caught. Only about half of anglers (56–63%) were successful at the start of the season when few walleye had yet been angled (0.0–0.1 proportion caught; Fig 3B). Trip success ranged from 64–86% successful across most of the range of the proportion of the population caught and then declined at the highest proportions captured (e.g., 42–50% successful at 140%– 150% of the population angled; Fig 3B).

Environmental factors that influenced walleye trip success were those generally associated with light intensity (i.e., diel period and solar radiation), as well as wind direction and lunar variables. We hypothesized that low light conditions would influence walleye behavior and as such anglers would be more likely to be success at periods of low light (i.e., diel periods of dawn and dusk, or at lower solar radiation values). The predicted odds of a successful trip was highest at dusk (0.47, 95% CI 0.33–0.63) followed by dawn (0.34, 95% CI 0.23–0.47), and then day (0.26, 95% CI 0.18–0.37). Anglers were observed to be about 10–15% more successful when fishing at dawn and dusk relative to the daytime period (Fig 2C). Mean daily solar radiation had a negative effect on walleye trip success. An angler’s odds of success were 0.44 (95% CI 0.28–0.58) at the lowest observed solar radiation 3 W/m^2^ declining to a predicted 0.26 (95% CI 0.14–0.41) probability of success at 570 W/m^2^ the maximum solar radiation value. The observed percent of successful trips tended to increase with decreasing solar intensity from about 42% successful at 425 W/m^2^ to 77% successful at 50 W/m^2^ (Fig 4A). However, sample sizes were low at the highest and lowest ends of the solar intensity values observed (e.g., < 25 W/m^2^ and ≥ 450 W/m^2^).

We hypothesized that wind factors, associated with storms and front activity, may influence walleye feeding behavior or angler behavior on the lake (e.g., where anglers chose to fish). Wind direction had high variable importance but wind speed and the wind speed and direction interaction did not (Table 1). The top model predicted a negative relationship between wind direction and the odds of a successful trip (Table 1). The observed data showed relatively higher trip success when peak wind was from an easterly direction (60°– 100°, range 74–76%). However, the effect size was considered very small (Cohen’s d < 0.20) and the observed trip success did not differ greatly across the range of wind direction (Fig 4B).

We hypothesized that lunar variables would influence walleye behavior and thus the probability of catching a walleye. Lunar position had a high variable importance weight and a small effect size (Table 1). The odds of a successful trip were predicted to be higher when the moon was underfoot (0.47, 95% CI 0.33–0.63) or overhead during a trip (0.41, 95% CI 0.28–0.56) relative to neither lunar passing occurring during a trip (0.34, 95% CI 0.23–0.47). Anglers were observed to be about 10–12% more likely to be successful when the moon was overhead or underfoot during their trip rather than when neither lunar event occurred during an angling event (Fig 2D). Lunar phase also had high variable importance (0.99, Table 1). The odds of a successful trip were highest during the gibbous phases (waxing gibbous 0.47, 95% CI 0.33–0.61; waning gibbous 0.45, 95% CI 0.32–0.59) and lowest during the quarter phases (last quarter phase 0.28, 95% CI 0.18–0.41; first quarter phase 0.34, 95% CI 0.23–0.48). The observed proportion of trips successful did not vary greatly among lunar phases but was relatively higher (71% successful) during the gibbous phases (Fig 5).

#### Catch rate

There was a total of 936 positive catch targeted walleye trips during 2003–2015. Initial model fit using log_e_(CPUE) showed a deviation from normal (Kolmogorov-Smirnov test p-value < 0.05; Fig 2 in S2 Appendix). Additional transformations were tested *post-hoc* for the positive truncated CPUE data (e.g., log10, square root, cubed root, Box-Cox transformation and Tukey’s lambda). The Tukey’s lambda power transformation (lambda = 0.02) provided a distribution closest to a normal transformation (i.e., Shapiro-Wilk normality test p-value = 0.03). Seven models were plausible (ΔAIC_c_ < 8.0). The top model provided a more reasonable fit relative to all other plausible models (ΔAIC_c_ differing by > 2.0; Table 1). The top model (ΔAIC_c_ 0.0) included the variables bait type, guide status, proportion of the population caught, diel period, air temperature, wind direction, and lunar position (Table 2 in S2 Appendix and Table 1). Variable weighting suggested that bait type, guide status, the proportion of the population caught and mean air temperature were of the highest relative importance (variable weight 1.0), followed by lunar position (variable weight 0.99), wind direction (variable weight 0.95), and diel period (variable weight 0.86, Table 1). The effect size of guide status, bait type, and the proportion of the population captured was considered small (Cohen’s d > 2.0 and < 4.0), and the effects of all other variables were classified as very small (Cohen’s d < 2.0; Table 1) [67].

Walleye CPUE for successful trips was significantly higher when using live bait relative to artificial bait only. Observed mean CPUE for successful trips was 1.32 walleye/h (95% CI 1.25–1.42) when using live bait and mean 0.68 walleye/h (95% CI 0.55–0.80) when using artificial bait (Fig 6A). The top model (after controlling for all other variables) predicted a mean CPUE for successful trips of 0.84 walleye/h (95% CI 0.78–0.91) when using artificial bait only and a mean CPUE for successful trips of 0.95 walleye/h (95% CI 0.91–0.99) when using live bait. Using artificial bait only when fishing for walleye was relatively uncommon. Out of all targeted walleye trips, there were a total of only 153 trips (7.6%) that used artificial bait only and 1,852 trips that used live bait (92.4%). The use of a guide resulted in a higher mean CPUE for successful trips (1.58 walleye/h, 95% CI 1.43–1.77) relative to successful unguided trips (1.22 walleye/h, 95% CI 1.13–1.32) in the observed data (Fig 6B). The top model predicted 0.90 walleye/h (95% CI 0.83–0.97) for successful guided trips and 0.84 walleye/h (95% CI 0.78–0.91) for successful unguided trips. The proportion of the walleye population captured had a negative effect on CPUE of successful trips. Walleye CPUE for successful trips was predicted at 0.91 walleye/h (95% CI 0.84–0.99) early in the season when few to no walleye had yet been angled. It declined to 0.74 walleye/h (95% CI 0.66–0.83) for successful trips that occurred when approximately 150% of the walleye population angled. Observed data showed similar CPUE for successful trips across the range of 0.0% to 100% of the population angled but then began to decline after an estimated 100% of the population had been caught (Fig 7A).

Walleye activity levels change in relation to air temperature as associated with seasonal behavioral changes (i.e., spring spawning behavior and fall increase in foraging behavior) as well as metabolic costs associated with foraging [29,30,51,53]. Thus, we hypothesized that angler catch rates would follow a similar pattern with known walleye activity changes associated with temperature and be higher at lower temperatures and decline at high temperatures (i.e., times of potential metabolic stress). Observed walleye CPUE for successful trips was higher and more variable at lower air temperatures < 10°C and generally declined as air temperature increased (Fig 7B). Predicted mean CPUE for successful trips declined with increasing air temperature, from a mean CPUE 0.91 walleye/h (95% CI 0.84–0.98) at temperatures ≤ 5°C to a mean of 0.78 walleye/h (95% CI 0.70–0.86) at temperatures ≥ 30°C.

Other environmental variables, including lunar position, wind direction and diel period, were weighted relatively high however their effect size was considered very small (Table 1). Similar to our hypotheses regarding light effects on angler trip success, we hypothesized that angler catch rates would be higher at times of lower light levels. Observed CPUE for successful trips was highest at dusk (1.47 walleye/h, 95% CI 1.21–1.76) relative to dawn (1.41 walleye/h, 95% CI 1.23–1.63) and day (1.23 walleye/h, 95% CI 1.15–1.33; Fig 6C). Predicted CPUE for successful trips did not differ much by diel period (predicted mean CPUE dusk 0.90 walleye/h, 95% CI 0.83–0.96; dawn 0.84 walleye/h, 95% CI 0.77–0.90; day 0.84 walleye/h, 95% CI 0.77–0.90). Solar radiation had low variable importance (Table 1). Thus, light effects were more apparent on walleye trip success rather than angler catch rate.

We hypothesized that lunar events (i.e., lunar phase and lunar position) would influence walleye behavior and thus, angler CPUE. Lunar position had relatively high variable importance but very small effect size (Table 1). Observed mean CPUE for successful trips by lunar position was highest when the moon was neither overhead nor underfoot (neither 1.35 walleye/h, 95% CI 1.25–1.45; overhead 1.14 walleye/h, 95% CI 0.99–1.31; underfoot 1.18 walleye/h, 95% CI 1.01–1.37; Fig 6D). There was little difference in the mean CPUE for successful trips among lunar position predicted by the top model (predicted mean CPUE neither 0.84 walleye/h, 95% CI 0.78–0.91; underfoot 0.80 walleye/h, 95% CI 0.75–0.89; overhead 0.80 walleye/h, 95% CI 0.73–0.87). Lunar phase had low variable importance (Table 1). Thus, lunar effects did have the same type of influence on CPUE as they appeared to have on walleye angler trip success.

We assumed storm and wind related variables would have similar effects on CPUE as they did on angler trip success. Wind direction had relatively high variable importance all other wind or storm related variables were of low importance (Table 1). The top model predicted a negative association between peak wind direction and catch rate (Table 1). However, the predicted mean CPUE for successful trips varied relatively little across the range of wind direction, e.g., a range of 0.82–0.84 walleye/h when winds were out of the north and north-west to a range of 0.85–0.86 walleye/h when winds were out of the south and south-east. The observed mean CPUE for successful trips was relatively stable across the range of wind direction values (Fig 7C).

### Muskellunge

#### Trip success

There was a total of 2,771 angling trips that targeted muskellunge only during 2003–2015. The probability of a successful muskellunge trip (i.e., catching at least one fish) during 2003–2015 was 0.18 (95% CI 0.17–0.19) and ranged from a low of 0.12 in 2009 to a high of 0.32 in 2003. There were eleven models that were considered plausible (ΔAIC_c_ ≤ 8.0) and the first six models did not differ appreciably from the top model (ΔAIC_c_ differed by ≤ 2.0; Table 3 in S2 Appendix and Table 2). The top model provided an adequate fit with no significant outliers, deviations or dispersion of the predicated values (Fig 3 in S2 Appendix). The variables bait type and lunar position had a variable weight of 1.0 (Table 2) meaning they were the only variables that were included in all weighted models. Other variables with relatively high importance weights included guide status, solar radiation, the proportion of the population caught, and wind direction (Table 2). All variables effects were considered very small (Cohen’s d < 0.2; Table 2).

**Table 2:** Muskellunge *Esox masquinongy* full hurdle model parameter results for trip success and positive truncated catch rate data (CPUE) for targeted muskellunge trips only from Escanaba Lake, WI during 2003–2015.

*Muskellunge trip success*								
Variable	Variable type	Coefficient (± SE)	Lower 95% CI	Upper 95% CI	z - value	p - value	Cohen’s d	Variable importance weight
Guide [Yes]	angler choice	0.36 (± 0.14)	0.08	0.64	2.51	0.012	0.10	0.999
Bait [Live]	angler choice	0.41 (± 0.14)	0.13	0.69	2.88	0.004	0.11	1.000
Trip density	angler	-0.09 (± 0.06)	-0.21	0.03	-1.48	0.138	-0.06	0.384
Proportion caught	population	-0.11 (± 0.07)	-0.24	0.03	-1.54	0.123	-0.06	0.957
Muskellunge density	population	0.15 (± 0.10)	-0.05	0.34	1.46	0.143	0.06	0.561
Barometric pressure	storm	0.06 (± 0.05)	-0.04	0.16	1.15	0.249	0.04	0.120
Precipitation	storm	0.01 (± 0.05)	-0.09	0.11	0.17	0.861	0.01	0.015
Wind speed	storm	0.03 (± 0.06)	-0.09	0.14	0.37	0.710	0.01	0.055
Wind direction	storm	-0.08 (± 0.05)	-0.03	0.18	1.44	0.150	0.05	0.877
Wind interaction	storm	-0.09 (± 0.05)	-0.19	0.01	-1.73	0.084	-0.07	0.055
Air temperature	seasonal	0.06 (± 0.07)	-0.07	0.19	0.86	0.390	0.03	0.210
Solar radiation	seasonal/light	-0.13 (± 0.07)	-0.26	0.00	-1.98	0.048	-0.08	0.989
Diel [Day]	light	-0.04 (± 0.13)	-0.30	0.22	-0.31	0.759	-0.01	0.720
Diel [Dusk]	light	0.68 (± 0.37)	-1.42	0.05	-1.83	0.068	-0.07	0.720
Lunar phase [Full]	lunar	0.19 (± 0.21)	-0.22	0.61	0.91	0.361	0.03	0.001
Lunar phase [Last quarter]	lunar	0.25 (± 0.21)	-0.21	0.60	0.94	0.348	0.04	0.001
Lunar phase [New]	lunar	0.02 (± 0.21)	-0.39	0.43	0.10	0.923	0.00	0.001
Lunar phase [Waning crescent]	lunar	0.19 (± 0.20)	-0.21	0.58	0.93	0.352	0.04	0.001
Lunar phase [Waning gibbous]	lunar	0.25 (± 0.21)	-0.17	0.67	1.19	0.235	0.05	0.001
Lunar phase [Waxing crescent]	lunar	0.07 (± 0.21)	-0.34	0.47	0.32	0.751	0.01	0.001
Lunar phase [Waxing gibbous]	lunar	0.18 (± 0.21)	-0.24	0.60	0.84	0.399	0.03	0.001
Lunar position [Overhead]	lunar	0.43 (± 0.14)	0.15	0.71	3.02	0.003	0.11	1.000
Lunar position [Underfoot]	lunar	0.35 (± 0.15)	0.05	0.65	2.26	0.024	0.09	1.000
*Muskellunge CPUE*								
Variable	Variable type	Coefficient (± SE)	Lower 95% CI	Upper 95% CI	z - value	p - value	Cohen’s d	Variable importance weight
Guide [Yes]	angler choice	-0.01 (± 0.07)	-0.16	0.13	-0.20	0.838	-0.01	0.03
Bait [Live]	angler choice	-0.12 (± 0.07)	-0.27	0.02	-1.71	0.088	-0.06	1.00
Trip density	angler	0.04 (± 0.03)	-0.02	0.10	1.41	0.158	0.05	0.58
Proportion captured	population	-0.04 (± 0.03)	-0.10	0.02	-1.22	0.221	-0.05	0.78
Muskellunge density	population	-0.01 (± 0.03)	-0.06	0.05	-0.32	0.750	-0.01	0.03
Barometric pressure	storm	0.01 (± 0.02)	-0.04	0.05	0.36	0.722	0.01	0.03
Precipitation	storm	0.06 (± 0.05)	-0.04	0.15	1.14	0.255	0.04	0.20
Wind speed	storm	-0.06 (± 0.03)	-0.12	-0.01	-2.18	0.029	-0.08	0.93
Wind direction	storm	-0.04 (± 0.03)	-0.09	0.01	-1.43	0.154	-0.05	0.36
Wind interaction	storm	-0.02 (± 0.03)	-0.07	0.03	-0.69	0.492	-0.03	0.05
Air temperature	seasonal	0.04 (± 0.03)	-0.02	0.11	1.26	0.206	0.05	0.97
Solar radiation	seasonal/light	0.03 (± 0.03)	-0.03	0.10	1.04	0.300	0.04	0.11
Diel [Day]	light	0.19 (± 0.07)	0.07	0.32	2.96	0.003	0.11	1.00
Diel [Dusk]	light	0.53 (± 0.20)	0.14	0.93	2.64	0.008	0.10	1.00
Lunar phase [Full]	lunar	0.12 (± 0.11)	-0.10	0.34	1.10	0.270	0.04	0.98
Lunar phase [Last quarter]	lunar	0.23 (± 0.11)	0.02	0.44	2.10	0.036	0.08	0.98
Lunar phase [New]	lunar	0.31 (± 0.11)	0.10	0.52	2.87	0.004	0.11	0.98
Lunar phase [Waning crescent]	lunar	0.17 (± 0.10)	-0.03	0.38	1.66	0.096	0.06	0.98
Lunar phase [Waning gibbous]	lunar	0.36 (± 0.11)	0.14	0.59	3.19	0.001	0.12	0.98
Lunar phase [Waxing crescent]	lunar	0.08 (± 0.11)	-0.12	0.29	0.79	0.432	0.03	0.98
Lunar phase [Waxing gibbous]	lunar	0.25 (± 0.11)	0.04	0.47	2.28	0.023	0.09	0.98
Lunar position [Overhead]	lunar	-0.16 (± 0.07)	-0.30	-0.02	-2.18	0.030	-0.08	1.00
Lunar position [Underfoot]	lunar	-0.39 (± 0.08)	-0.55	-0.23	-4.83	0.000	-0.18	1.00

Muskellunge *Esox masquinongy* full hurdle model parameter estimates ± SE and 95% confidence intervals, z-value, p-value, effect size (Cohen’s d) with 95% confidence intervals, and variable importance weighting derived from AIC_c_ model selection procedures for trip success and positive truncated catch rate data (CPUE), for targeted muskellunge trips only from Escanaba Lake, WI during 2003–2015. Variable type indicates the categorical designation for each variable used in model selection.

Similar to walleye, angler specific variables that had a high variable weighting included the use of live bait and the use of a guide (Table 2). However, the relative effect of these variables were not as strong as those observed for walleye. Anglers that used live bait when fishing for muskellunge had a higher predicted odds of success 0.20 (95% CI 0.14–0.28) relative to those using artificial bait (predicted odds of success 0.14, 95% CI 0.10–0.19). Twenty-five percent of observed trips during 2003–2015 were successful compared to those that used artificial bait only which were 16% successful (Fig 8A). Twenty-four percent of trips that used a guide when fishing for muskellunge were successful relative to those that did not use a guide (17% of trips successful; Fig 8B). Model results predicted that the odds of a successful trip when using a guide was 0.18 (95% CI 0.13–0.26) relative to 0.14 for non-guided trips (95% CI 0.10–0.19).

**Fig 9:**
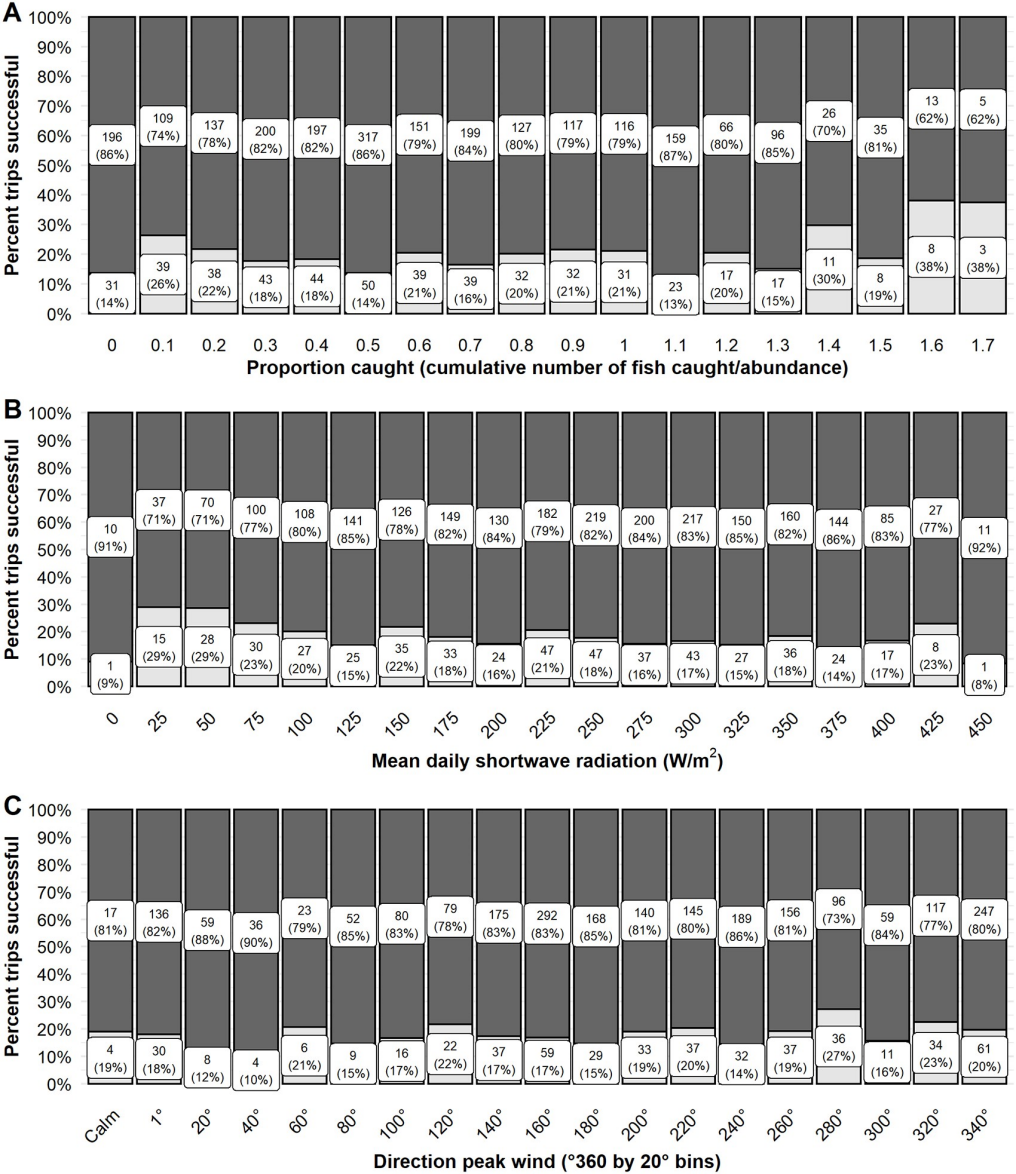
The observed proportion of trips successful for muskellunge. The observed proportion of successful trips for model variables the proportion of the population caught (A), solar radiation (W/m^2^; B), and wind direction (°360; C) on Escanaba. Lake, WI, USA. The observed proportion of trips successful are indicated by the lower, light grey, portion of the column. Unsuccessful trips are indicated by the dark grey, upper portion of the column. The percent successful or unsuccessful is indicated by the label within each section. The sample size of trips included in each category is indicated below the bar.

We hypothesized that muskellunge may experience learning behavior or lure avoidance and thus, trip success would be influenced by the cumulative proportion of fish captured each year. The proportion of the muskellunge population caught had relatively high variable weighting but the effect size was considered very small (Table 2). The top model predicted a negative relationship between trip success and the probability of a successful trip (Table 2). The predicted odds of a successful trip did not differ greatly across the range of the proportion of the population caught. The predicted odds of a successful muskellunge trip was 0.14 (95% CI 0.09–0.21) early in the season when zero to low numbers of the population had been caught. Whereas the predicted odds of a successful muskellunge trip was 0.12 (95% CI 0.07–0.20) at the upper end of the spectrum, 150% of the population had been caught (i.e., each muskellunge in the population may have been caught at least once or some caught multiple times). Alternatively, the observed data showed a relatively similar trip success rates across most of the range of the proportion of the population caught (Fig 9A). Observed trip success was relatively higher at the highest observed proportions of the population caught (38% success at > 160% of the population captured) however, the sample sizes at this upper range of the data were relatively low compared to the sample sizes available across the rest of the range of this variable (Fig 9A).

Environmental factors that had the highest variable importance for trip success were lunar position, mean daily solar radiation, and resultant wind direction (Table 2). We hypothesized that muskellunge activity levels would be influenced by lunar events which would then be reflected in angler trip success rates. An angler’s predicted odds of a successful muskellunge trip were 0.19 (95% CI 0.13–0.27) when the moon was overhead and 0.18 (95% CI 0.12–0.26) when the moon was underfoot. When neither lunar event occurred during a trip the odds of success were 0.13 (95% CI 0.09–0.18). Twenty-three percent of observed trips were successful when the moon was either overhead or underfoot during a fishing trip on Escanaba Lake relative to trips that did not encompass either of these lunar transition periods of which 16% were successful (Fig 8C).

We hypothesized that muskellunge would have a positive association with light variables due to observed increases in activity associated with changes in prey fish activity during the day. However, similar to walleye, solar radiation had a negative influence on muskellunge trip success and diel period had lower relative importance (< 0.75; Table 2). The predicted odds of a successful trip decreased from a high of 0.17 (95% CI 0.11–0.25) at solar radiation < 25 W/m^2^ to a predicted odds of success of 0.11 (95% CI 0.07–0.16) at solar radiation > 450 W/m^2^. Observed muskellunge trip success was relatively stable across the range of solar radiation values (e.g.,14–23% of trips were successful at solar values 75–425 W/m^2^; Fig 9B). There was a slightly higher success rate observed, 29%, at 25–50 W/m^2^ (Fig 9B). Sample size was low at the highest and lowest ends of the solar radiation spectrum.

We hypothesized that storm related effects, including wind, would impact muskellunge activity levels as has been reported anecdotally by anglers. Wind direction had relatively high variable importance relative to other variables (≥ 0.85; Table 2). However, its effect size was very small and no additional wind or storm variables were identified as important. The odds of a successful muskellunge trip were predicted to increase with increasing wind degree direction (Table 2). Observed data suggested that trip success was highest (27% of trips successful) when winds were out of the WNW (280°– 299°; Fig 9C). However, muskellunge trip success rate did not differ greatly across the range of wind direction.

#### Catch rate

Total muskellunge catch was low with the majority of successful trips only catching one fish (83.5%). However, 14.2% of successful trips caught two muskellunge, 1.7% caught three, and 0.4% four, so multiple catch trips were observed over time. The maximum number of muskellunge angled in one trip was five, which occurred once over the duration of the dataset (0.1%). There was a total of 505 successful muskellunge angling trips on Escanaba Lake during 2003–2015. There were 11 plausible models (ΔAIC_c_ < 8.0) and there were four models did not differ appreciably from the top model (ΔAIC_c_ differed by < 2.0; Table 4 in S2 Appendix). The top model fit reasonably well with no significant outliers, deviations or dispersion of the predicted values (Fig 4 in S2 Appendix). Bait type, diel period and lunar position had the highest variable importance weighting (variable importance weight = 1.0) followed by lunar phase, air temperature and wind speed (Table 2). All variables effects were considered very small (Cohen’s d < 0.2; Table 2) [67].

**Fig 10:**
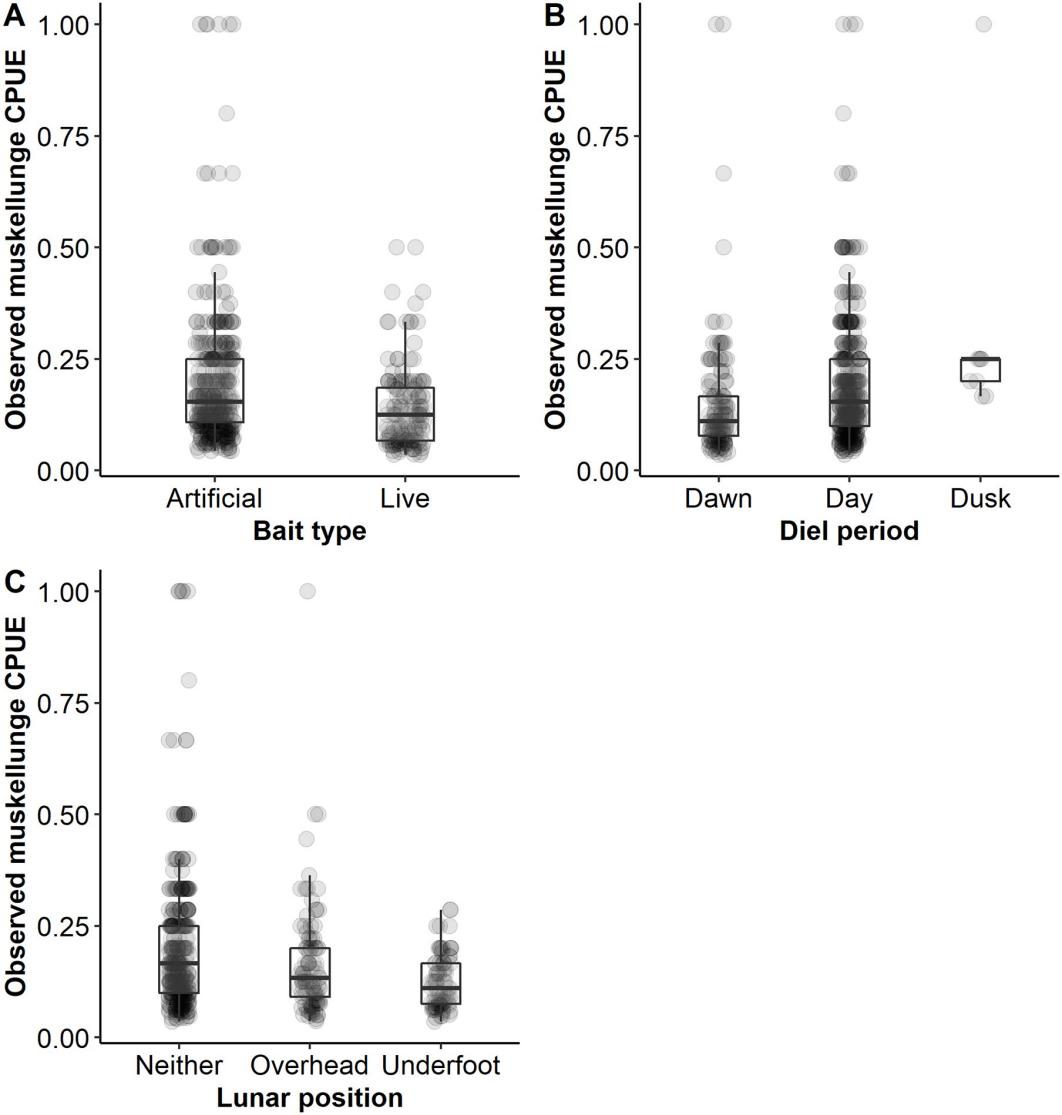
Observed catch rate for positive truncated muskellunge trips in relation to angler and population variables. The observed catch rate (CPUE) from positive truncated catch trips for model variables bait type (A), diel period (B) and lunar position (C) on Escanaba Lake, WI, USA. Trends in the observed catch rate for continuous variables are indicated with box plots and jittered points. Box plots represent the median catch rate (middle bar) and upper and lower quartiles (boundaries of the box).

Angler choice of bait type influenced muskellunge CPUE. Opposite of what was observed for walleye anglers on Escanaba lake, most muskellunge anglers (80%, n = 2213) used artificial bait only as opposed to live bait (20%, n = 558). The observed mean CPUE for successful trips using live bait was 0.14 muskellunge/h (95% CI 0.13–0.15) and 0.20 muskellunge/h (95% CI 0.19–0.22) for successful trips using artificial bait only (Fig 10A). Model predicted mean CPUE did not differ greatly by bait type. Predicted CPUE for success trips was 0.12 (95% CI 0.10–0.15) for artificial bait users and 0.11 (95% CI 0.09–0.13) for live bait users.

Light metric diel period had high variable importance but solar radiation did not (Table 2). Successful muskellunge fishing trips at dusk were uncommon (4%, n = 103) and there were only nine successful trips at dusk in the positive truncated dataset used in the model. Observed mean muskellunge CPUE for successful dawn trips was 0.15 muskellunge/h (95% CI 0.13–0.18, n = 132), successful day trips 0.19 muskellunge/h (95% CI 0.18–0.20, n = 364), and successful dusk trips muskellunge/h 0.30 (95% CI 0.20–0.49, n = 9; Fig 10B). The model predicted a similar relationship with predicted mean CPUE of 0.12 muskellunge/h (95% CI 0.10–0.15) for successful trips at dawn, 0.15 muskellunge/h for successful trips at daytime (95% CI 0.12–0.17), and 0.21 muskellunge/h for successful trips at dusk (95% CI 0.14–0.31; Fig 10B).

**Fig 11:**
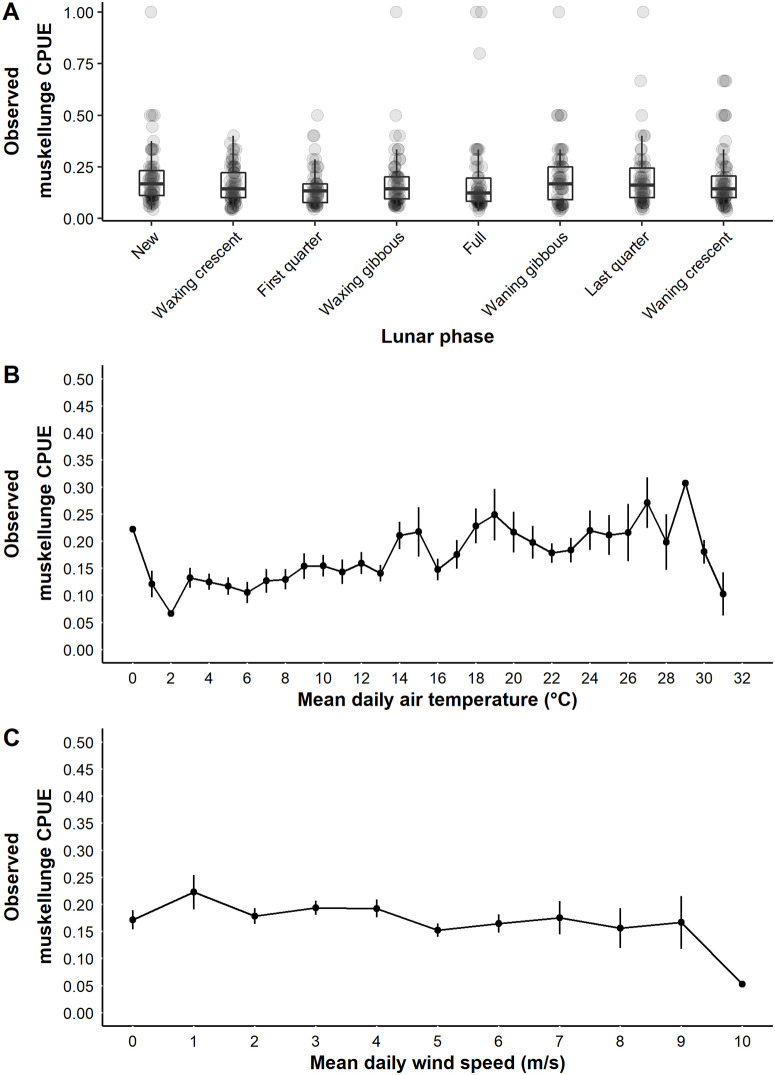
Observed catch rate for positive truncated muskellunge trips in relation to angler and population variables. The observed catch rate (CPUE) from positive truncated catch trips for model variables lunar phase (A), mean air temperature (°C; B), and mean daily wind speed (m/s; C) on Escanaba Lake, WI, USA. Trends in the observed catch rate for continuous variables are indicated with box plots and jittered points or for continuous variables the observed mean catch rates are indicated by the solid black point with error bars ± SE. Box plots represent the median catch rate (middle bar) and upper and lower quartiles (boundaries of the box).

Both lunar variables, position and phase, had high variable importance (Table 2). Lunar position was included in all weighted models but had very low effect size (Table 2). Observed mean CPUE for successful muskellunge trips was highest when neither lunar event occurred during a fishing trip, 0.20 muskellunge/h (95% CI 0.19–0.22, n = 324; Fig 10C). By comparison, the observed mean CPUE for successful trips when the moon was in the overhead position was 0.17 muskellunge/h (95% CI 0.14–0.19, n = 101) and the observed mean CPUE for successful trips in the underfoot position was 0.12 muskellunge/h (95% CI 0.11–014, n = 80; Fig 10C). Predicted results followed a similar pattern whereby predicted mean CPUE for successful trips was 0.12 muskellunge/h (95% CI 0.10–0.15) when neither lunar passing event occurred during the trip, relative to 0.11 muskellunge/h (95% CI 0.08–0.13) and 0.08 muskellunge/h (95% CI 0.06–0.10) for successful trips that occurred when the moon was overhead and underfoot, respectively. Lunar phase also had high relative importance but a low effect size (Table 2). Observed mean CPUE for successful trips was lowest during the first quarter phase (mean 0.15 muskellunge/h, 95% CI 0.13–0.18) and the waxing crescent phase (mean 0.16 muskellunge/h, 95% CI 0.14–0.19) following the new moon, and highest for successful trips that occurred during the waning gibbous and new moon phases, 0.21 muskellunge/h (95% 0.16–0.24) and 0.20 muskellunge/h (95% CI 0.17–0.24), respectively (Fig 11A). Predicted mean CPUE for successful trips followed the same pattern and ranged from a low of 0.12 muskellunge/h (95% CI 0.10–0.15) during the first quarter moon to a high of 0.17 muskellunge/h (95% CI 0.14–0.17) for successful trips that occurred during the waning gibbous phase.

We hypothesize that air temperature would be negatively related to muskellunge CPUE for successful trips. Specifically, we assumed that CPUE would be higher in the spring and fall in association with spawning and fall foraging activity and then decline as summer temperatures limited activity due to physiological stress. However, observed muskellunge CPUE for successful trips tended to increase with mean daily air temperature and then began to decline at air temperatures > 29°C (Fig 11B). Predicted CPUE for successful trips ranged from a low of 0.10 muskellunge/h at temperatures ≤ 5°C and increased to 0.14 muskellunge/h at air temperatures ≥ 25°C.

Log_e_(CPUE) of successful trips was negatively related to mean daily wind speed however the relationship was relatively weak (Table 2). Observed mean CPUE of successful trips appeared relatively stable ranging between 0.15 muskellunge/h and about 0.20 muskellunge/h CPUE except for one low CPUE observation at the highest observed wind speed (10 m/s; Fig 11C). Predicted mean CPUE for successful trips ranged from 0.15 muskellunge/h (0.12–0.19) at relatively calm wind speeds (≤ 1 m/s) and declined to 0.09 muskellunge/h (95% CI 0.07–0.13) at the maximum wind speeds observed (≥ 9 m/s).

## Discussion

Walleye and muskellunge are two highly sought-after species with angler tactics and fish vulnerability potentially influencing angling trip success and catch rates. Our results demonstrated that angler choice metrics (i.e., use of a guide or choice of bait), the proportion of the population that has previously been angled, lunar metrics (i.e., lunar position and phase), light metrics (i.e., diel period and solar radiation), air temperature and wind metrics (i.e., wind direction and speed) can contribute to successful walleye and muskellunge angling trips and higher walleye catch rates in Escanaba Lake. These angler-specific results suggest angling vulnerability in individual fish is influenced by a fish’s internal state, encounter probability, environmental conditions, and outfitted angling gear. For example, a guide with knowledge and experience of fish behavior, habitat, and past angling success would likely increase angler encounter rates, thereby increasing angling vulnerability of walleye and muskellunge. Daily trip density may increase with favorable environmental conditions and/or catch rates leading to higher overall catch rates and trip success. Increased trip densities may be related to anglers’ perceptions of best fishing times and conditions. The type of angling gear used would ultimately determine a fish’s decision to strike [10] and establish a successful catch. Since gear is usually selective for morphological traits [10], our results further provide evidence of increased angling vulnerability since bait choice contributed to trip success and catch rates of walleye and muskellunge in Escanaba Lake. However, the effect of bait type differed between species and may have been influenced in part by sample size. The use of live bait dominated walleye trips and walleye anglers were found to have higher success and catch rate when using live bait reflecting the trend that was observed by Bailey et al. (2019) [16]. On the other hand, the use of artificial bait dominated angler choice for muskellunge anglers. Bait choice had a mixed influence on muskellunge trips where the use of live bait positively influenced the probability of catching at least one muskellunge (e.g., trip success), but successful anglers had higher predicted catch rates when using artificial bait only. The catch of multiple muskellunge on any given angling trip was low on Escanaba Lake during the study period. Multiple catch trips did occur but it’s likely that the low occurrence of catch rate > 1.0 and a high proportion of anglers using artificial only bait for muskellunge could explain the mixed result between bait influence on trip success versus catch rate. Future research is needed to determine the relative influence of skewed bait type choices on trips success and catch rates.

In addition to the positive influence of angler tactics on muskellunge trip success, results also indicated that the proportion of the population caught tended to have a negative effect on angling vulnerability. Our results indicated that as a larger proportion of the walleye or muskellunge population was caught and released over the season in Escanaba Lake, angler trip success and CPUE was predicted to decline. This suggests that greater angling exposure may have resulted in learned avoidance behavior in both muskellunge and walleye and consequently contributed to angling vulnerability. The decision for an individual fish to strike, and potentially contribute to a successful catch, is determined by a fishes’ previous experience and threat perception [12]. Boldness and aggression [20,21], stress responsiveness [22], and memory retention [10,23–25] influence angling vulnerability. However, it is important to note that in most cases the observed trip success data did not display a clearly negative trend and similar trip success rates occurred across a wide range of the proportion of the population caught and released for both walleye and muskellunge. In this study we were unable to further evaluate the influence of angler metrics and fish population metrics due to lack of data regarding angler fishing location, walleye and muskellunge habitat use, individual fish vulnerability, or associations between prey availability and fish density. Future research could improve upon this study by considering these factors in association with angler choice metrics and environmental variables.

The fishing community has often attributed catch rate variations to changes in environmental conditions [26], which are also likely correlated with underlying mechanisms that function in a fishes’ angling vulnerability [26,27,68]. Quantifying fish vulnerability to angling requires identifying environmental variation in catch rates, which has rarely been researched in freshwater recreational angling events [27]. However, our results validate that several specific environmental factors affect angling trip success and catch rates in walleye and muskellunge suggesting an influence of these factors on vulnerability of fish to recreational angling. Walleye and muskellunge trip success and CPUE for successful walleye trips on Escanaba Lake generally increased as light conditions decreased (i.e., decreasing solar radiation and the diel periods of dawn or dusk relative to daytime). Muskellunge CPUE results on the other hand had mixed effects with a higher predicted catch rates at dusk and day time relative to dawn. Fish experience a light environment that varies temporally and spatially [26] with light availability affecting peak activity and foraging times in many different species [10,27,69,70]. Walleye possess a retina with a tapetum lucidum that allows for scotopic vision and efficient foraging during low light environments [30] which may increase prey encounters, alter reactivity to fishing gear from reduced water transparency [26], and increase vulnerability to lures and angling [27]. Muskellunge on the other hand do not possess the same low light adaptations as walleye [53] and differences in trip success and catch rate may not be related to light intensity itself but rather other factors such as prey activity. Prey species such as bluegills *Lepomis macrochirus* typically feed during diurnal periods [71–73], while yellow perch activity has been found to be highest during the day [74], simultaneously increasing their vulnerability to predation and potentially triggering muskellunge movement during this period [54].

Multiple environmental factors related to seasonal changes in environmental conditions (i.e., air temperature, solar radiation, and diel period) contributed to walleye and muskellunge trip success and catch rates. Thus, changes in angling vulnerability may be occurring due to factors associated with spring spawning behavior via spring warming rates and relative changes in light availability and day length triggers. During spawning activities, individuals congregate in the littoral zone of the lake and fish may be more active than at other times of the year cruising the area for suitable habitat and mates [29,30,51,53] making them more vulnerable to anglers. Additionally, Escanaba Lake experiences low levels of ice fishing effort in most years, thus the early spring season after ice-out also coincides with the end of a period of reduced angling pressure. Fish may potentially display decreased lure avoidance in the spring relative to the summer or fall seasons when angling effort is higher and more consistent over time [12,49]. Environmental factors, such as water temperature, control the biological processes affecting energy and feeding requirements of a fish [10]. Hunger induced by spawning metabolic demands and decreased perception from minimal ice fishing pressure may contribute to increased angling vulnerability during the spring months in Escanaba Lake.

The lunar cycle generates cues recognized by many different species [75] and associated changes in lunar illumination are believed to have a pronounced effect on predator-prey dynamics [34,35,76,77]. Our results illustrated that walleye trip success and muskellunge catch rate on Escanaba Lake was positively influenced by specific lunar phases (i.e., waxing and waning gibbous, full and new moons). Lunar position (i.e., moon overhead or underfoot) particularly influenced the odds of success when fishing for walleye or muskellunge. Few studies have documented lunar effects on walleye, with the exception stating that recently hatched walleye inhabiting rivers increased their drift rates in response to moon stages [30]. Predation is affected by the optical conditions in aquatic environments [78,79], with environmental conditions affecting the sensory capabilities of predators and prey [77,80–82]. Past studies have determined that diel, wind, and lunar effects on vertical migrations of zooplankton were likely driven by food availability, predators, and physiological costs [83–86] and it may be possible that walleye and muskellunge prey on fish that react and feed on zooplankton during lunar migrations. However, the observed response to lunar phase and position via illumination as a mechanism of maximizing foraging effectiveness may inadequately describe walleye and muskellunge responses in Escanaba Lake. The NHFRA designates fishing hours and there is no legal fishing allowed at night, so in this case lunar illumination would not have a direct effect on angling trip success. Our study observed only trips that occurred during the daytime and crepuscular hours, suggesting the potential influence of other phenomena associated with the lunar cycle rather than lunar illumination. The lunar cycle is an environmental factor that generates cues recognized by many different species and includes changes not just in light intensity, but also geomagnetism and gravity [75]. Vinson and Angradi (2014) [36] demonstrated that muskellunge catch increased during new and full moon periods and our results also indicated a lunar phase effect on muskellunge where catch rates were relatively higher after the full moon and during the new moon. Electromagnetic effects have been found to produce larger disturbances in solar-terrestrial interactions at higher latitudes [56] and this corresponds with the Vinson and Angradi (2014) [36] observation of a stronger lunar effect on muskellunge caught at high latitudes (>48°N; e.g., Escanaba Lake, WI 46°N). Muskellunge possess a well-developed lateral line system and use somatosensory cues to relay critical information when pursuing prey [87]; however, it is currently unknown whether muskellunge possess the ability to detect geomagnetic changes—particularly electrical phenomena—that occur in response to lunar variation. Tom Gelb, a longtime muskellunge angler in Vilas County, WI and the surrounding region, kept meticulous records of his fishing trips for decades including catch and environmental conditions. In his 2012 book, *Musky Strategy*, Gelb suggested that trophy muskellunge success was higher in the hour before and after moon rise, moonset, and when the moon was underfoot (e.g., day times during the full moon and night times during the new moon). Similar to the Gelb (2012) muskellunge observation, trips that experienced a moon overhead or underfoot occurrence had higher odds of success for both walleye and muskellunge on Escanaba Lake. Our results suggest that lunar cycles (phase and position) have an influence on muskellunge and walleye angling vulnerability, and while the exact mechanisms that induce these differences is unknown, it is likely that they are related to geomagnetic or gravitational influences on behavior rather than lunar illumination alone.

Wind factors affected walleye and muskellunge angling vulnerability on Escanaba Lake. In particular, wind direction was a highly weighted variable for both walleye and muskellunge trip success as well as walleye CPUE, while wind speed was a highly weighted variable for muskellunge catch rate. Interestingly, neither model included both wind factors together or the wind speed–wind direction interaction term. Strong winds or wind fetch induce wave action and can increase turbidity within an aquatic environment [88], affecting foraging and feeding activity of many fishes [89] by potentially influencing encounter rates from incurred velocity differences [90–92]. Since muskellunge are visual predators [53,93], their ability to effectively detect and capture prey may be optimized on calmer days with reduced turbidity which may subsequently induce angling vulnerability due to increased encounter rates. Additionally, anglers may identify wind speed, direction, and the resulting wave action with a change in fish behavior and choose windward lake areas or identify these as hazardous conditions and move to leeward areas. Ultimately, we do not record angler catch location or general fishing locations as part of the NHFRA creel, so we are unable to determine whether wind effects were influencing muskellunge and walleye vulnerability via changes in fish behavior, angler behavior, or both. Future studies investigating environmental factors and catch rates could improve upon this research by incorporating more information on angling location in relation to environmental factors.

Many anglers have speculated that changes in weather, often those associated with fronts, affect angling catch for a variety of freshwater fish species. In our study, we found significant effects of solar radiation, air temperatures, and wind direction on walleye angling (i.e., trip success and/or catch rates) and effects of solar radiation, air temperature, wind speed, and wind direction on muskellunge angling (i.e., trip success and/or catch rates). However, some trends of these identified significant factors did not align with known weather changes associated with front activity or storms. For example, muskellunge catch rates were higher on calmer days. Additionally, large changes in barometric pressure and precipitation are associated with frontal weather and yet these environmental factors were not highly weighted in any analysis. Since we did not consider variable interactions within the model framework, we recommend additional research to determine whether the influence of wind, solar radiation, air temperature variables are signifying effects of frontal activity or influencing walleye and muskellunge angling in different ways.

Trip-specific angler variables tended to be the most highly weighted factors influencing trip success and catch rates for walleye and muskellunge. This suggests that angler knowledge of a particular species and waterbody (e.g., guides and repeat visitors to the lake) as well as angler behavior (i.e., amount of effort per trip, bait type choice) had the strongest influence on whether or not a fishing trip would be successful or multiple fish would be caught in one fishing trip. We did not report correlations between angler-specific variables and environmental variables, but it is likely that angler behavior differs in relation to environmental factors. For example, knowledgeable anglers may be more likely to fish during prime daily periods like dawn or dusk and prime seasonal periods like spring or fall. Experienced anglers may also be more likely to spend more time angling per trip (i.e., increased effort) relative to a casual or novice angler. The summer period tends to attract more tourists to the area, more first-time visitors to the lake, and more children angling while on summer vacation. Factors such as angler experience (i.e., age and how many times they have fished Escanaba Lake) may certainly influence trip success and catch. These experience factors could be more heavily weighted to summer periods when the highest amount of trips and angling effort occurs, which could influence the significant environmental effects observed on trip success and catch rates. For example, skilled anglers that fished during summer vacation may contribute to increased trip success and catch rates during periods of time with increased mean solar radiation and mean air temperature. Angler age demographics and residency are included in NHFRA creel permit information; however, individual angler identification, which would be needed to determine the number of times an angler has fished the lake, is not currently in the database. Future research could examine the influence of angler age demographics and angler experience (i.e., number of visits to Escanaba Lake) to test whether these factors are correlated with environmental variables and trip success and catch rates.

The results of our study suggest that walleye and muskellunge angling vulnerability is influenced by multiple environmental factors as well as angler-specific metrics and fish population metrics. Generally, walleye anglers have the highest expectations and often associate a positive fishing experience with greater catch rates [94]. Muskellunge are an elusive, highly sought-after species that construe a toilsome angling event, thereby stimulating an angler’s desire to discern specific conditions implicative of increased odds and catch rates [53,93]. Scientific literature testing for the effect of environmental factors on freshwater angler catch is limited [27,36], but was recently acknowledged as a pertinent recreational fisheries topic in a study that surveyed experts and stakeholders [4]. Therefore, management goals tend to focus on balancing fish populations, creating satisfactory catch rates, and providing opportunities to catch trophy-sized fish [8,9]. As such, a better understanding of the influences of environmental factors on angling vulnerability is an important component of fisheries management [5–7]. Our results suggest that angler-specific variables, light, temperature, lunar, and weather conditions influence species-specific angling vulnerability for walleye and muskellunge, which is important information for managers to sustain these fisheries and meet angler desires.

## Supporting information

S1 Appendix Contains all the supporting figures investigating temporal trends in fish population and angler variables. (DOCX)

S2 Appendix Contains all the supporting information regarding model fit and variable effect sizes. (DOCX)
