# Supplementary material for: Angler and environmental influences on walleye Sander vitreus and muskellunge Esox masquinongy angler catch in Escanaba Lake, Wisconsin 2003–2015
Source: PLoS One. 2021 Sep 30;16(9):e0257882. doi: 10.1371/journal.pone.0257882 (PMC8483380; doi:10.1371/journal.pone.0257882)
Supplement: S1 Appendix — (DOCX) [file pone.0257882.s001.docx]

**Supplement 1: Temporal trends in continuous variables**

*Temporal trends in fish population and angler variables*


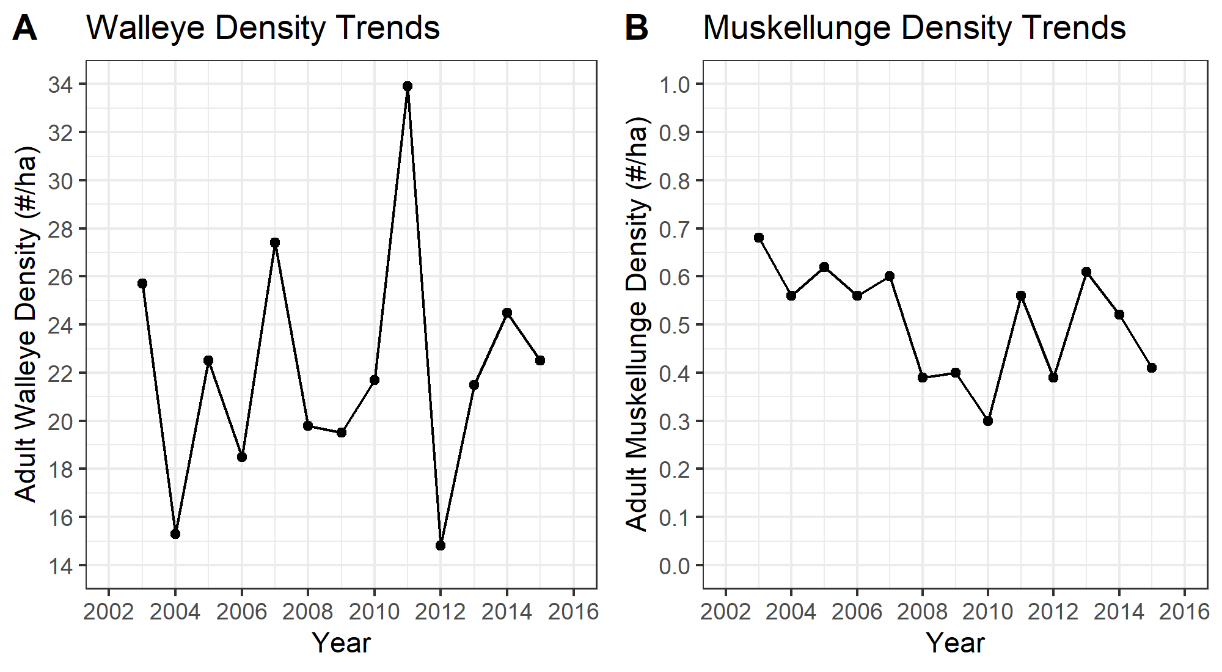


Fig 1. A) Walleye and B) muskellunge density did not differ significantly during 2003-2015 on Escanaba Lake, Wisconsin, USA.


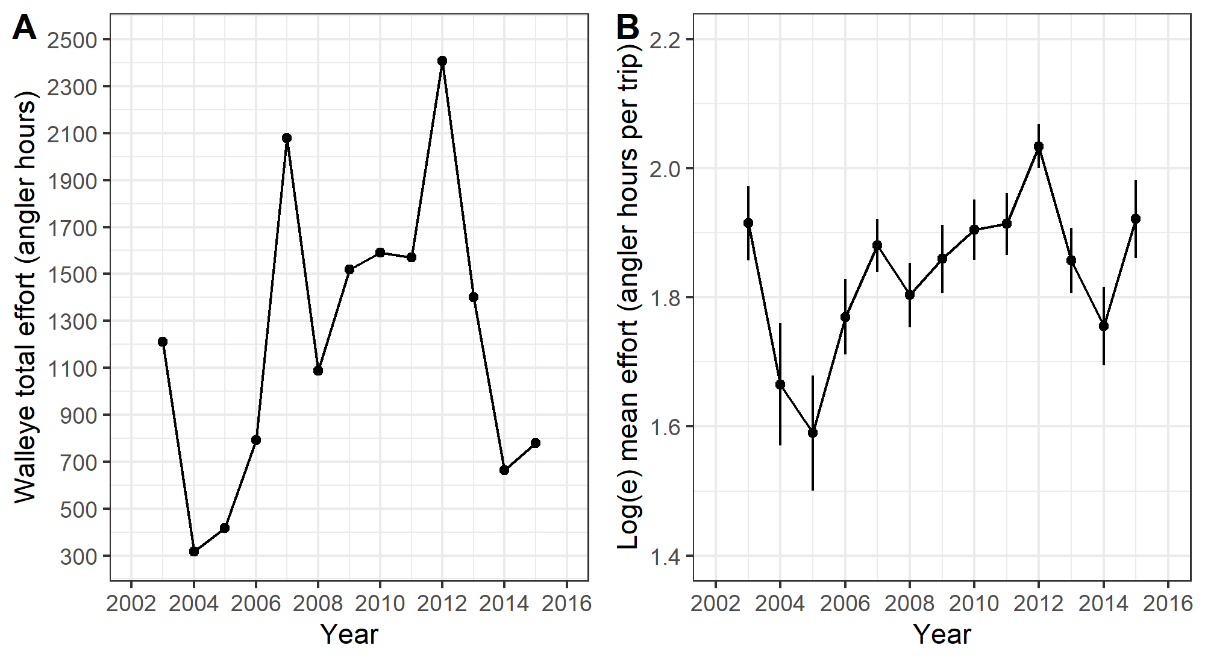


Fig 2. A) Walleye total annual effort, B) walleye log_e_ mean effort per trip did not differ during 2003-2015 on Escanaba Lake, Wisconsin, USA.


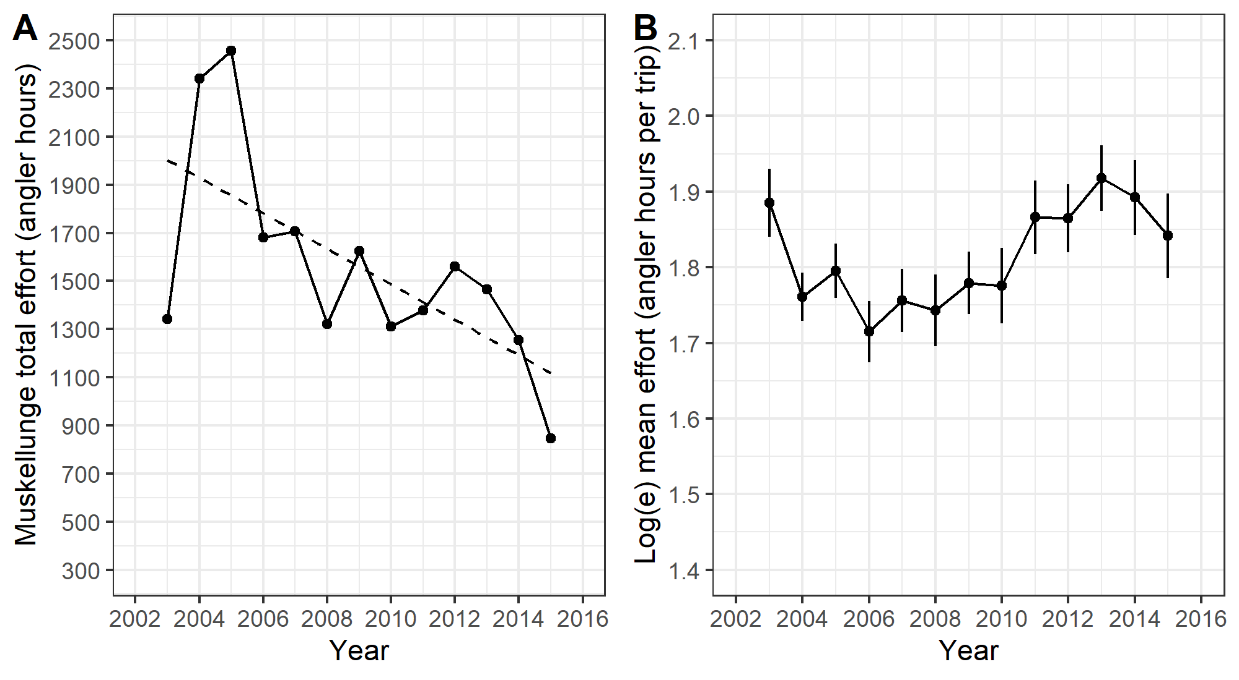


Fig 3. A) muskellunge total annual effort declined significantly (F_1,11_ = 8.5, p-value = 0.01) and B) muskellunge log_e_ mean effort per trip did not differ, during 2003-2015 on Escanaba Lake, Wisconsin, USA.


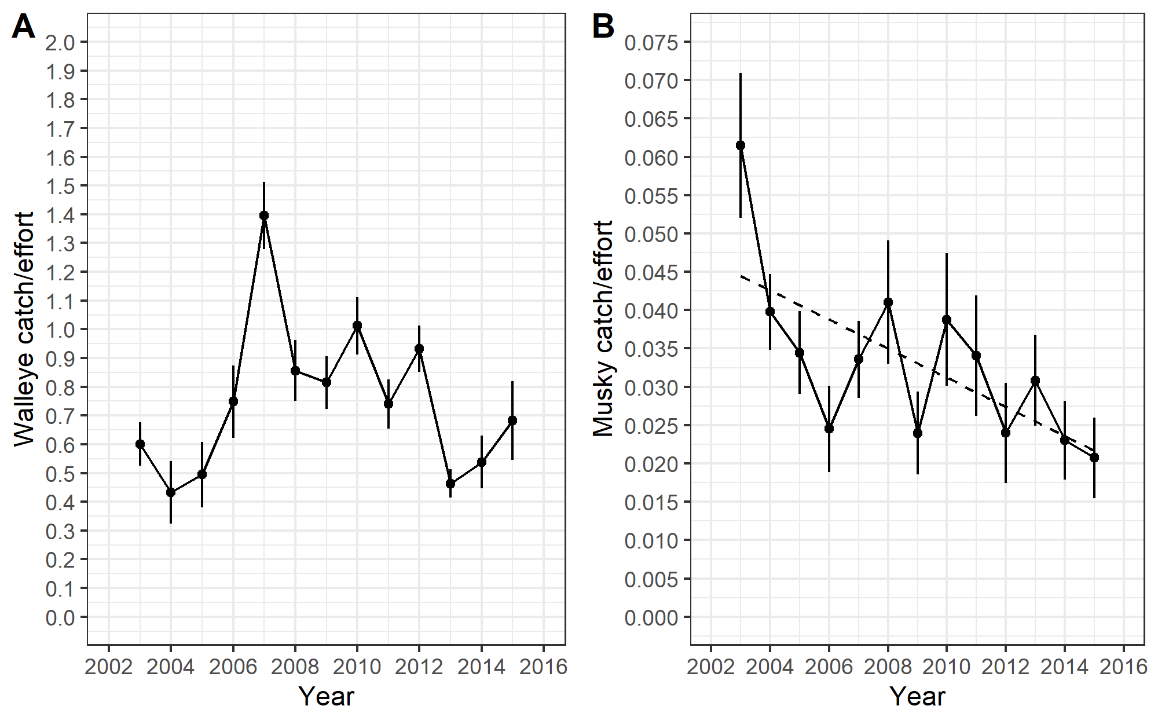


Fig 4. A) Walleye and B) muskellunge catch per unit effort (total fish per angler hour) over time. Natural log walleye catch per unit effort did not differ during 2003-2015. Natural log muskellunge catch per unit effort significantly declined (F_1,2769_ = 15.7, p-value < 0.001).

*Temporal trends in weather variables*


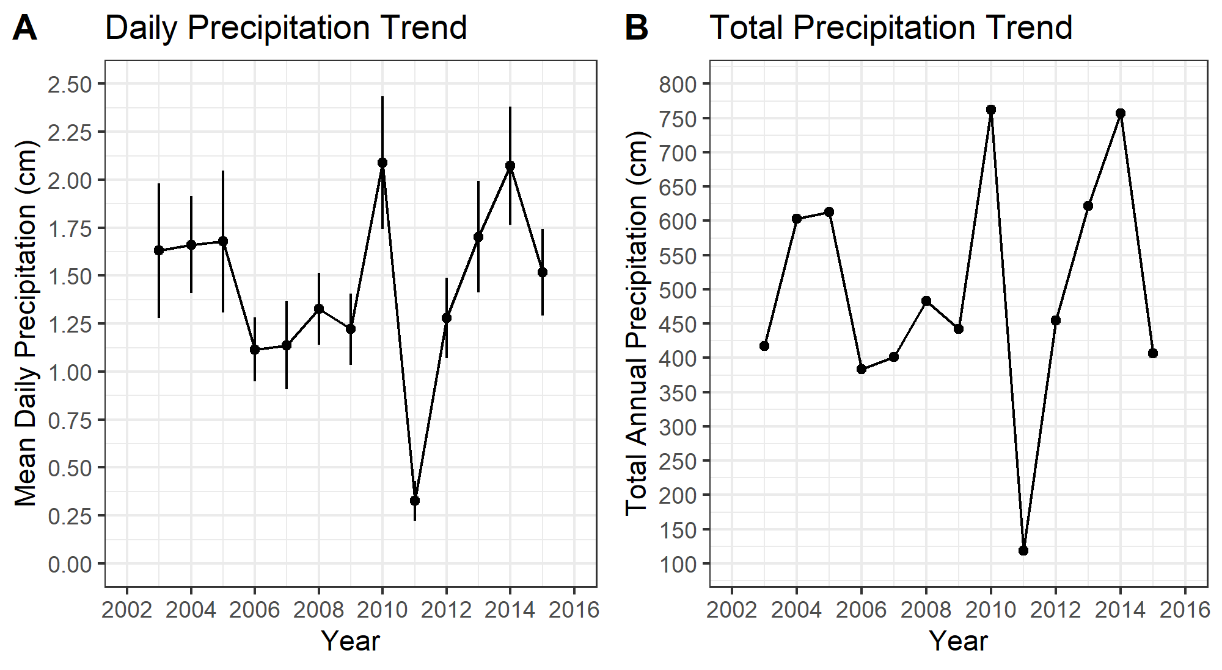


Fig 5. A) Mean daily precipitation (cm) by year and B) total annual precipitation (cm) did not differ during 2003-2015 on Escanaba Lake, WI, USA. Hourly precipitation data was acquired from the North Temperate Lakes Long-Term Ecological Research meteorological dataset field site location at Noble F. Lee Municipal airport in Woodruff, WI, USA (18 km from Escanaba Lake).


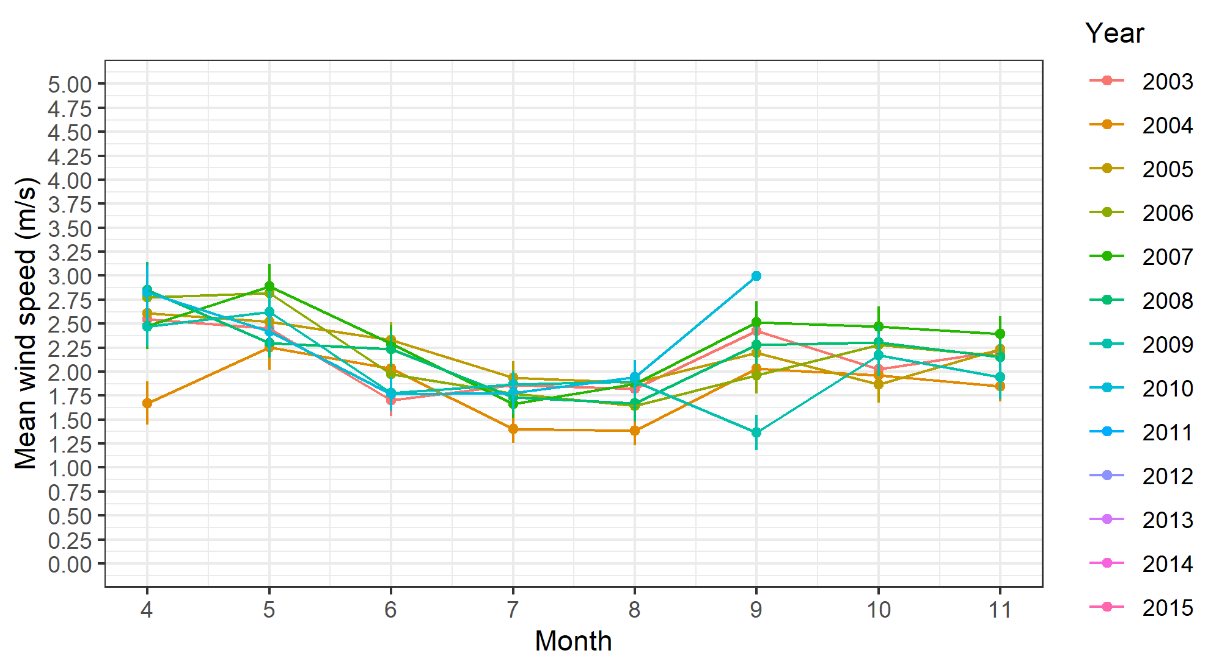


Fig 6. Mean monthly wind speed (m/s) did not differ among years during 2003-2015 on Escanaba Lake, WI, USA.


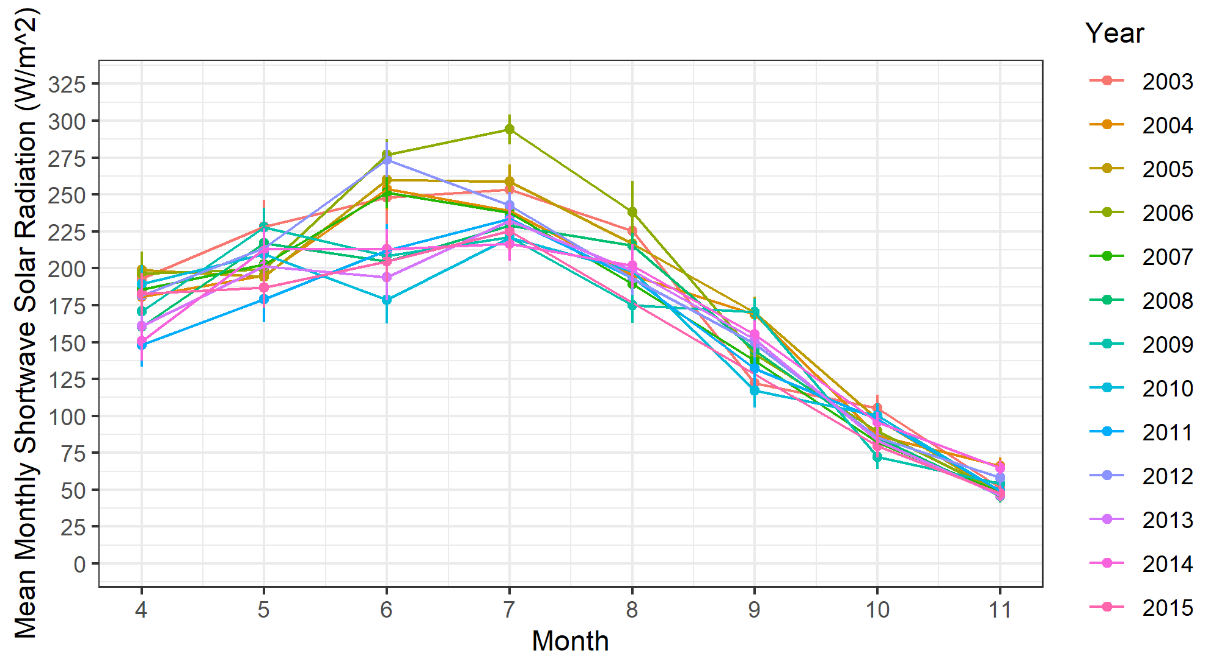


Fig 7. Mean monthly solar radiation (W/m^2^) did not differ among years during 2003-2015 on Escanaba Lake, WI, USA.


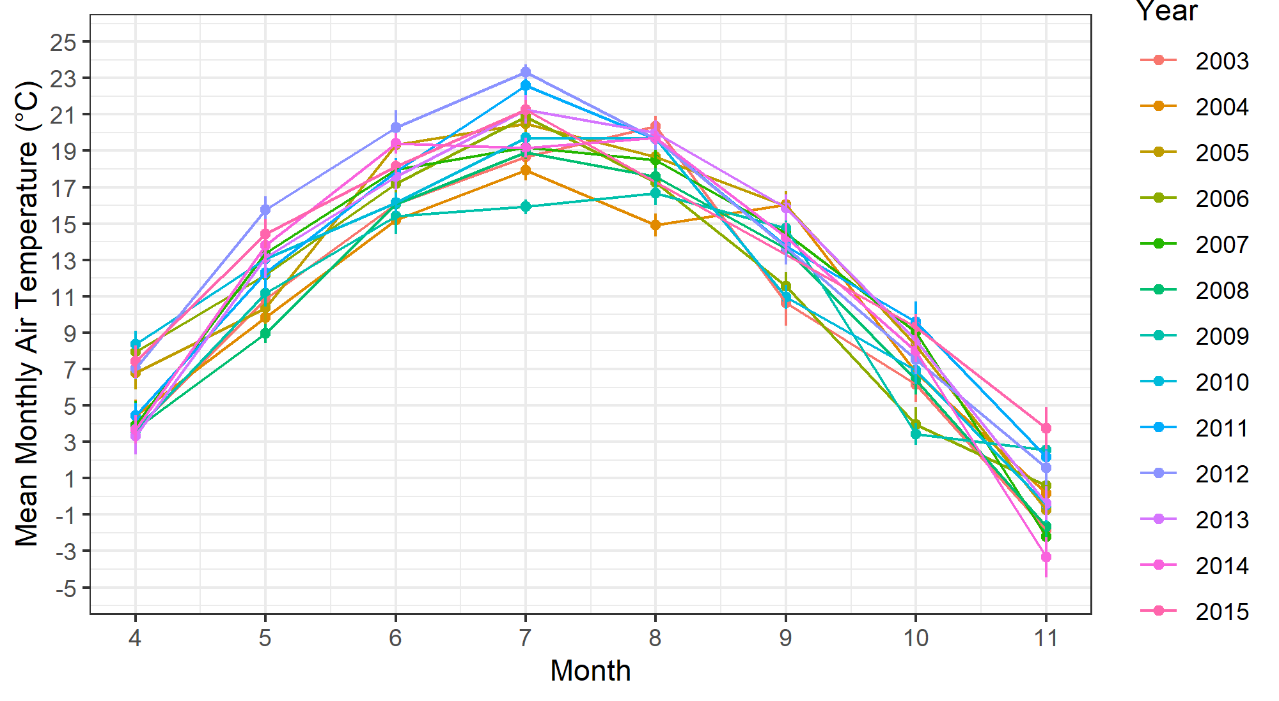


Fig 8. Mean monthly air temperature (°C) did not differ among years during 2003-2015 on Escanaba Lake, WI, USA.
