# Supplementary material for: Angler and environmental influences on walleye Sander vitreus and muskellunge Esox masquinongy angler catch in Escanaba Lake, Wisconsin 2003–2015
Source: PLoS One. 2021 Sep 30;16(9):e0257882. doi: 10.1371/journal.pone.0257882 (PMC8483380; doi:10.1371/journal.pone.0257882)
Supplement: S2 Appendix — (DOCX) [file pone.0257882.s002.docx]

**Supplement 2: Model fit and variable effect sizes**

I) *Walleye trip success AICc model selection table, top model fit and variable effect sizes.*

Table 1. AICc model selection results for walleye *Sander vitreus* trip success including model variables, model degrees of freedom, log likelihood (LL), AICc value, ΔAICc, model weight ($w_{t}$), model rank, and log likelihood based R^2^ value (i.e., pseudo R^2^). Model variables included: bait type used (i.e., live or artificial; bait), whether or not a trip was guided (guide), daily trip density (i.e., boats per ha; trip density), the cumulative proportion of the walleye population caught and released (proportion caught), adult walleye density (i.e., number of adult walleye/ha; walleye density), daily barometric pressure trend (barometric pressure), cumulative precipitation during trip (precipitation), mean daily solar radiation (solar radiation), daily mean wind speed (wind speed), direction of peak wind (wind direction), wind speed and direction interaction term (wind speed:wind direction), diel period (i.e., dawn, day or dusk; diel period), lunar phase (i.e., 8 factor characterization of moon phase; lunar phase), lunar position (i.e., overhead, underfoot or neither; lunar position), and daily mean air temperature (air temperature). If an interaction term is specified in the table then three terms were included in the model, one for each variable and one for the variable interaction. In all models including the null model year was included as a random effect.

| **Fixed Effects** | **df** | **LL** | **AIC_c_** | **ΔAIC_c_** | **w_t_** | **R^2^** |
| --- | --- | --- | --- | --- | --- | --- |
| ~ bait + guide + trip density + proportion caught + barometric pressure + diel period + lunar phase + lunar position + solar radiation + wind direction | 20 | -1172.41 | 2385.24 | 0.00 | 0.372 | 0.119 |
| ~ bait + guide + trip density + proportion caught + diel + lunar phase + lunar position + solar radiation + wind direction | 19 | -1173.67 | 2385.71 | 0.48 | 0.293 | 0.118 |
| ~ bait + guide + trip density + proportion caught + barometric pressure + diel period + lunar phase + lunar position + solar radiation + wind direction + wind speed | 21 | -1172.16 | 2386.78 | 1.54 | 0.172 | 0.119 |
| ~ bait + guide + trip density + proportion caught + barometric pressure + diel period + lunar phase + lunar position + wind direction + wind speed + wind speed:wind direction | 22 | -1171.92 | 2388.36 | 3.12 | 0.078 | 0.119 |
| ~ bait + guide + trip density + proportion caught + walleye density + air temperature + barometric pressure + diel period + lunar phase + lunar position + precipitation + solar radiation + wind direction | 23 | -1172.24 | 2391.04 | 5.80 | 0.020 | 0.119 |
| ~ bait + guide + trip density + proportion caught + walleye density + air temperature + barometric pressure + diel period + lunar phase + lunar position + precipitation + solar radiation + wind direction + wind speed + wind speed:wind direction | 24 | -1171.81 | 2392.23 | 6.99 | 0.011 | 0.119 |
| ~ bait + guide + trip density + proportion caught + walleye density + air temperature + barometric pressure + diel period + lunar phase + lunar position + precipitation + solar radiation + wind direction + wind speed + wind speed:wind direction | 24 | -1171.82 | 2392.25 | 7.01 | 0.011 | 0.119 |
| ~ bait + guide + trip density + proportion caught + walleye density + air temperature + barometric pressure + diel period + lunar phase + lunar position + solar radiation + wind direction + wind speed + wind speed:wind direction | 24 | -1171.9 | 2392.41 | 7.17 | 0.010 | 0.119 |
| ~ bait + guide + trip density + proportion caught + walleye density + air temperature + barometric pressure + diel period + lunar phase + lunar position + precipitation + solar radiation + wind direction + wind speed | 24 | -1172.01 | 2392.62 | 7.38 | 0.009 | 0.119 |
| ~ bait + guide + trip density + proportion caught + diel period + lunar position + solar radiation + wind direction | 12 | -1184.39 | 2392.93 | 7.69 | 0.008 | 0.108 |
| ~ bait + guide + trip density + proportion caught + walleye density + air temperature + barometric pressure + diel period + lunar phase + lunar position + precipitation + solar radiation + wind direction + wind speed + wind speed:wind direction | 25 | -1171.81 | 2394.27 | 9.03 | 0.004 | 0.119 |
| ~ bait + guide + trip density + proportion caught + walleye density + air temperature + diel period + lunar phase + lunar position + precipitation + solar radiation + wind direction + wind speed + wind speed:wind direction | 24 | -1173.01 | 2394.63 | 9.39 | 0.003 | 0.118 |
| ~ bait + guide + trip density + proportion caught + diel period + lunar position + solar radiation | 1 | -1186.74 | 2395.61 | 10.37 | 0.002 | 0.106 |
| ~ bait + guide + trip density + proportion caught + walleye density + air temperature + barometric pressure + diel period + lunar phase + lunar position + precipitation + wind direction + wind speed + wind speed:wind direction | 24 | -1173.91 | 2396.43 | 11.19 | 0.001 | 0.117 |
| ~ bait + guide + trip density + proportion caught + walleye density + air temperature + diel period + lunar phase + lunar position | 19 | -1179.19 | 2396.76 | 11.52 | 0.001 | 0.113 |
| ~ bait + guide + trip density + proportion caught + walleye density + air temperature + barometric pressure + diel period + lunar phase + lunar position + precipitation + solar radiation + wind speed | 23 | -1175.31 | 2397.18 | 11.94 | 0.001 | 0.116 |
| ~ bait + guide + trip density + air temperature + barometic pressure + diel period + lunar phase + lunar position + precipitation + solar radiation + wind direction + wind speed + wind speed:wind direction | 23 | -1175.4 | 2397.35 | 12.11 | 0.001 | 0.116 |
| ~ bait + guide + trip density + walleye density + air temperature + barometric pressure + diel period + lunar phase + lunar position + precipitation + solar radiation + wind direction + wind speed + wind speed:wind direction | 24 | -1175.2 | 2399.01 | 13.77 | 0.000 | 0.116 |
| ~ bait + guide + trip density + proportion caught + walleye density + air temperature + barometric pressure + diel period + lunar position + precipitation + solar radiation + wind direction + wind speed + wind speed:wind direction | 18 | -1182.7 | 2401.75 | 16.51 | 0.000 | 0.110 |
| ~ bait + guide + trip density + proportion caught + walleye density + air temperature + barometric pressure + diel period + lunar phase + precipitation + solar radiation + wind direction + wind speed + wind speed:wind direction | 23 | -1178.77 | 2404.1 | 18.86 | 0.000 | 0.113 |
| ~ bait + guide + trip density + proportion caught + diel period + solar radiation | 9 | -1194.56 | 2407.22 | 21.98 | 0.000 | 0.099 |
| ~ bait + guide + proportion caught + walleye density + air temperature + barometric pressure + diel period + lunar phase + lunar position + precipitation + solar radiation + wind direction + wind speed + wind speed:wind direction | 24 | -1181.18 | 2410.97 | 25.74 | 0.000 | 0.111 |
| ~ bait + guide + trip density + proportion caught + walleye density + air temperature + barometric pressure + diel period + precipitation + solar radiation + wind direction + wind speed + wind speed:wind direction | 16 | -1189.97 | 2412.21 | 26.97 | 0.000 | 0.103 |
| ~ bait + guide + trip density + proportion caught + diel period | 8 | -1198.46 | 2413 | 27.76 | 0.000 | 0.096 |
| ~ bait + guide + trip density + proportion caught + walleye density + air temperature + barometric pressure + lunar phase + lunar position + precipitation + solar radiation + wind direction + wind speed + wind speed:wind direction | 23 | -1184.34 | 2415.24 | 30.00 | 0.000 | 0.108 |
| ~ bait + guide + trip density + proportion caught + walleye density + air temperature + barometric pressure + lunar phase + lunar position + precipitation + wind direction + wind speed + wind speed:wind direction | 22 | -1185.53 | 2415.56 | 30.32 | 0.000 | 0.107 |
| ~ guide + trip density + proportion caught + walleye density + air temperature + barometric pressure + diel period + lunar phase + lunar position + precipitation + solar radiation + wind direction + wind speed + wind speed:wind direction | 24 | -1185.99 | 2420.58 | 35.34 | 0.000 | 0.107 |
| ~ bait + guide + trip density + proportion caught | 6 | -1209.59 | 2431.22 | 45.98 | 0.000 | 0.086 |
| ~ bait + guide + trip density | 5 | -1215.72 | 2441.47 | 56.23 | 0.000 | 0.080 |
| ~ bait + guide | 4 | -1223.37 | 2454.76 | 69.52 | 0.000 | 0.073 |
| ~ guide | 3 | -1239.83 | 2485.67 | 100.43 | 0.000 | 0.058 |
| ~ bait + trip density + proportion caught + walleye density + air temperature + barometric pressure + diel period + lunar phase + lunar position + precipitation + solar radiation + wind direction + wind speed + wind speed:wind direction | 24 | -1223.89 | 2496.38 | 111.14 | 0.000 | 0.072 |
| ~ trip density + proportion caught + walleye density + air temperature + barometric pressure + diel period + lunar phase + lunar position + precipitation + solar radiation + wind direction + wind speed + wind speed:wind direction | 23 | -1242.9 | 2532.37 | 147.13 | 0.000 | 0.055 |
| ~ proportion caught + walleye density + air temperature + barometric pressure + diel + lunar phase + lunar position + precipitation + solar radiation + wind speed:wind direction + wind speed + wind speed:wind direction | 22 | -1249.16 | 2542.83 | 157.59 | 0.000 | 0.049 |
| ~ null | 2 | -1299.202 | 2602.405 | 217.17 | 0.000 | 0.000 |


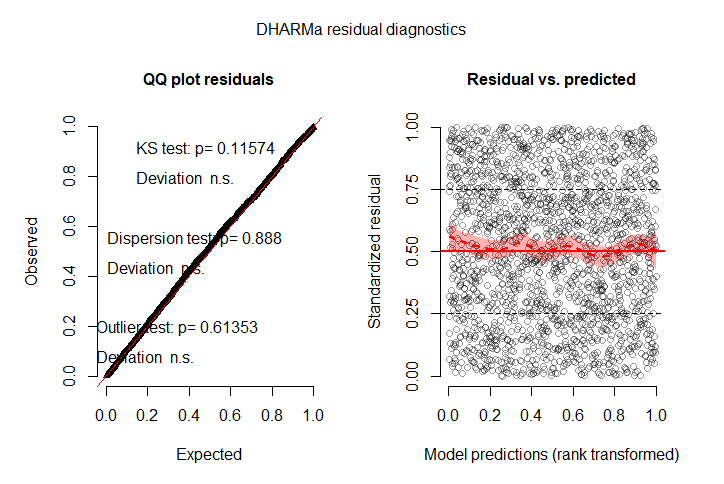


Fig 1. QQ-plot and residual versus predicted plot for the top ranked model exploring factors that affect walleye trip success on Escanaba Lake, WI, USA.

II) *Walleye catch per unit effort AICc model selection table, top model fit and variable effect sizes.*

Table 2. AICc model selection results for walleye *Sander vitreus* positive truncated catch per unit effort data including model variables, model degrees of freedom (df), log likelihood (LL), AICc value, ΔAICc, model weight ($w_{t}$), model rank

and log likelihood based R^2^ value (i.e., pseudo R^2^). Model variables included: bait type used (i.e., live or artificial; bait), whether or not a trip was guided (guide), daily trip density (i.e., boats per ha; trip density), the cumulative proportion of the walleye population caught and released (proportion caught), adult walleye density (i.e., number of adult walleye/ha; walleye density), daily barometric pressure trend (barometric pressure), cumulative precipitation during trip (precipitation), mean daily solar radiation (solar radiation), daily mean wind speed (wind speed), direction of peak wind (wind direction), wind speed and direction interaction term (wind speed:wind direction), diel period (i.e., dawn, day or dusk; diel period), lunar phase (i.e., 8 factor characterization of moon phase; lunar phase), lunar position (i.e., overhead, underfoot or neither; lunar position), and daily mean air temperature (air temperature). If an interaction term is specified in the table then three terms were included in the model, one for each variable and one for the variable interaction. In all models including the null model year was included as a random effect.

| **Fixed Effects** | **df** | **LL** | **AIC_c_** | **ΔAIC_c_** | **w_t_** | **R^2^** |
| --- | --- | --- | --- | --- | --- | --- |
| ~ bait + guide + proportion captured + diel period + lunar position + air temperature + wind direction | 12 | 237.5 | -450.75 | 0.00 | 0.719 | 0.071 |
| ~ bait + guide + proportion captured + lunar position + air temperature + wind direction | 10 | 233.5 | -446.82 | 3.93 | 0.101 | 0.065 |
| ~ bait + guide + proportion captured + lunar position + air temperature | 9 | 231.48 | -444.81 | 5.94 | 0.037 | 0.062 |
| ~ bait + guide + trip density + proportion captured + diel period + lunar phase + lunar position + air temperature + wind direction | 20 | 242.65 | -444.64 | 6.12 | 0.034 | 0.078 |
| ~ bait + guide + trip density + proportion captured + diel period + lunar phase + lunar position + air temperature + solar radiation + wind direction | 21 | 243.55 | -444.38 | 6.38 | 0.030 | 0.079 |
| ~ bait + guide + proportion captured + diel period + lunar phase + lunar position + air temperature + wind direction | 19 | 241.3 | -444.01 | 6.74 | 0.025 | 0.076 |
| ~ bait + guide + trip density + proportion captured + walleye density + diel period + barometric pressure + lunar position + precipitation + air temperature + solar radiation + wind direction + wind speed + wind speed * wind direction | 19 | 240.92 | -443.23 | 7.52 | 0.017 | 0.075 |
| ~ bait + guide + trip density + proportion captured + diel period + lunar phase + lunar position + precipitation + air temperature + solar radiation + wind direction | 22 | 243.89 | -442.98 | 7.77 | 0.015 | 0.080 |
| ~ bait + guide + trip density + proportion captured + walleye density + diel period + lunar phase + lunar position + air temperature + solar radiation | 21 | 242.45 | -442.16 | 8.59 | 0.010 | 0.078 |
| ~ bait + guide + proportion captured + air temperature | 7 | 227.03 | -439.98 | 10.77 | 0.003 | 0.055 |
| ~ bait + guide + trip density + proportion captured + walleye density + diel period + barometric pressure + precipitation + air temperature + solar radiation + wind direction + wind speed + wind speed * wind direction | 17 | 237.04 | -439.6 | 11.16 | 0.003 | 0.070 |
| ~ bait + guide + trip density + proportion captured + diel period + lunar phase + lunar position + precipitation + air temperature + solar radiation + wind direction + wind speed + wind speed * wind direction | 24 | 244.04 | -439.13 | 11.63 | 0.002 | 0.080 |
| ~ bait + guide + trip density + proportion captured + walleye density + diel period + barometric pressure + lunar phase + lunar position + precipitation + air temperature + solar radiation + wind direction | 24 | 243.9 | -438.85 | 11.91 | 0.002 | 0.080 |
| ~ bait + guide + trip density + proportion captured + diel period + barometric pressure + lunar phase + lunar position + precipitation + air temperature + solar radiation + wind direction + wind speed + wind speed*wind direction | 25 | 244.05 | -437.06 | 13.69 | 0.001 | 0.080 |
| ~ bait + guide + trip density + proportion captured + walleye density + diel period + lunar phase + lunar position + precipitation + air temperature + solar radiation + wind direction + wind speed + wind speed * wind direction | 25 | 244.04 | -437.05 | 13.70 | 0.001 | 0.080 |
| ~ bait + guide + trip density + proportion captured + walleye density + diel period + barometric pressure + lunar phase + lunar position + precipitation + air temperature + solar radiation + wind speed | 24 | 242.84 | -436.73 | 14.03 | 0.001 | 0.078 |
| ~ bait + guide + trip density + proportion captured + walleye density + diel period + barometric pressure + lunar phase + lunar position + air temperature + solar radiation + wind direction + wind speed + wind speed * wind direction | 25 | 243.66 | -436.28 | 14.47 | 0.001 | 0.079 |
| ~ bait + guide + trip density + proportion captured + walleye density + diel period + barometric pressure + lunar phase + lunar position + precipitation + air temperature + wind direction + wind speed + wind speed * wind direction | 25 | 243.2 | -435.37 | 15.39 | 0.000 | 0.079 |
| ~ bait + guide + trip density + proportion captured + walleye density + diel period + barometric pressure + lunar phase + lunar position + precipitation + air temperature + solar radiation + wind direction + wind speed + wind speed * wind direction | 26 | 244.05 | -434.98 | 15.77 | 0.000 | 0.080 |
| ~ bait + guide + proportion captured + walleye density + diel period + barometric pressure + lunar phase + lunar position + precipitation + air temperature + solar radiation + wind direction + wind speed + wind speed * wind direction | 25 | 242.32 | -433.61 | 17.15 | 0.000 | 0.077 |
| ~ bait + guide + trip density + proportion captured + walleye density + diel period + barometric pressure + lunar phase + precipitation + air temperature + wind direction + wind speed + wind speed * wind direction | 24 | 240.81 | -432.67 | 18.09 | 0.000 | 0.075 |
| ~ bait + guide + trip density + proportion captured + walleye density + barometric pressure + lunar phase + lunar position + precipitation + air temperature + solar radiation + wind direction + wind speed + wind speed * wind direction | 24 | 239.87 | -430.8 | 19.96 | 0.000 | 0.074 |
| ~ bait + guide + proportion captured | 6 | 221.38 | -430.69 | 20.06 | 0.000 | 0.047 |
| ~ bait + guide + trip density + proportion captured + walleye density + diel period + barometric pressure + lunar phase + lunar position + precipitation + solar radiation + wind direction + wind speed + wind speed * wind direction | 25 | 240.5 | -429.97 | 20.79 | 0.000 | 0.075 |
| ~ bait + guide + trip density + diel period + barometric pressure + lunar phase + lunar position + precipitation + air temperature + solar radiation + wind direction + wind speed + wind speed * wind direction | 24 | 237.35 | -425.75 | 25.01 | 0.000 | 0.070 |
| ~ bait + guide + trip density + walleye density + diel period + barometric pressure + lunar phase + lunar position + precipitation + air temperature + solar radiation + wind direction + wind speed + wind speed * wind direction | 25 | 237.43 | -423.84 | 26.92 | 0.000 | 0.070 |
| ~ guide + trip density + proportion captured + walleye density + diel period + barometric pressure + lunar phase + lunar position + precipitation + air temperature + solar radiation + wind direction + wind speed + wind speed * wind direction | 25 | 236.65 | -422.26 | 28.49 | 0.000 | 0.069 |
| ~ guide + proportion captured | 5 | 214.01 | -417.98 | 32.78 | 0.000 | 0.036 |
| ~ bait + trip density + proportion captured + walleye density + diel period + barometric pressure + lunar phase + lunar position + precipitation + air temperature + solar radiation + wind direction + wind speed + wind speed * wind direction | 25 | 233.72 | -416.41 | 34.35 | 0.000 | 0.065 |
| ~ bait + trip density + proportion captured + walleye density + diel period + barometric pressure + lunar phase + lunar position + precipitation + solar radiation + wind direction + wind speed + wind speed * wind direction | 24 | 230.09 | -411.23 | 39.52 | 0.000 | 0.060 |
| ~ proportion captured | 4 | 205.62 | -403.21 | 47.54 | 0.000 | 0.023 |
| ~ proportion cpatured + walleye density + diel period + barometric pressure + lunar phase + lunar position + precipitation + air temperature + solar radiation + wind direction + wind speed + wind speed * wind direction | 23 | 224.54 | -402.2 | 48.55 | 0.000 | 0.052 |
| ~ trip density + proportion captured + walleye density + diel period + barometric pressure + lunar phase + lunar position + precipitation + air temperature + solar radiation + wind direction + wind speed + wind speed * wind direction | 24 | 225.19 | -401.43 | 49.32 | 0.000 | 0.053 |
| ~ null | 3 | 190.4 | -374.79 | 75.96 | 0.000 | 0.000 |


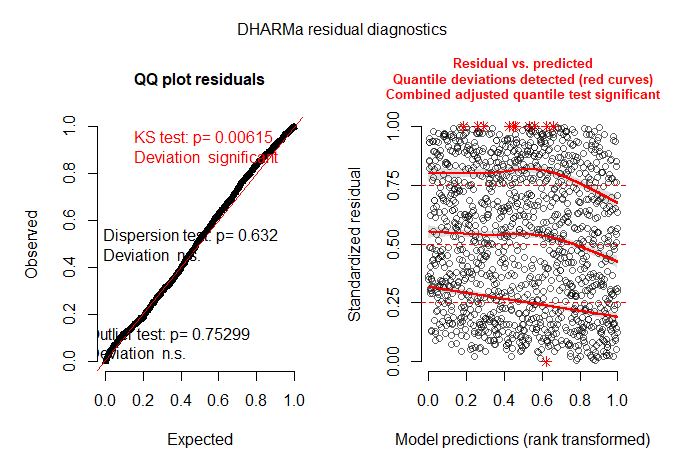


Fig 3. QQ-plot and residual versus predicted plot for the top ranked model exploring factors that affect walleye catch rate on Escanaba Lake, WI, USA.

III) *Muskellunge trip success AICc model selection table, top model fit and variable effect sizes.*

Table 3. AICc model selection results for muskellunge *Esox masquinongy* trip success including model variables, model degrees of freedom, log likelihood (LL), AICc value, ΔAICc, model weight ($w_{t}$), model rank and log likelihood based R^2^ value (i.e., pseudo R^2^). Model variables included: bait type used (i.e., live or artificial; bait), whether or not a trip was guided (guide), daily trip density (i.e., boats per ha; trip density), the cumulative proportion of the walleye population caught and released (proportion caught), adult muskellunge density (i.e., number of muskellunge ≥ 30 inches /ha; walleye density), daily barometric pressure trend (barometric pressure), cumulative precipitation during trip (precipitation), mean daily solar radiation (solar radiation), daily mean wind speed (wind speed), direction of peak wind (wind direction), wind speed and direction interaction term (wind speed:wind direction), diel period (i.e., dawn, day or dusk; diel period), lunar phase (i.e., 8 factor characterization of moon phase; lunar phase), lunar position (i.e., overhead, underfoot or neither; lunar position), and daily mean air temperature (air temperature). If an interaction term is specified in the table then three terms were included in the model, one for each variable and one for the variable interaction. In all models including the null model year was included as a random effect.

| **Fixed Effects** | **df** | **LL** | **AIC_c_** | **ΔAIC_c_** | **w_t_** | **R^2^** |
| --- | --- | --- | --- | --- | --- | --- |
| ~ bait + guide + proportion caught + muskellunge density + diel period + lunar position + solar radiation + wind direction | 12 | -1278.8 | 2581.67 | 0.00 | 0.177 | 0.022 |
| ~ bait + guide + trip density + proportion caught + muskellunge density + diel period + lunar position + solar radiation + wind direction | 13 | -1277.8 | 2581.70 | 0.03 | 0.174 | 0.022 |
| ~ bait + guide + proportion caught + diel period + lunar position + solar radiation + wind direction | 11 | -1279.9 | 2581.88 | 0.22 | 0.159 | 0.021 |
| ~ bait + guide + proportion caught + lunar position + solar radiation + wind direction | 9 | -1281.9 | 2581.91 | 0.24 | 0.157 | 0.020 |
| ~ bait + guide + trip density + proportion caught + muskellunge density + air temperature + diel period + lunar position + solar radiation + wind direction | 14 | -1277.4 | 2583.03 | 1.36 | 0.090 | 0.023 |
| ~ bait + guide + proportion caught + lunar position + solar radiation | 8 | -1283.6 | 2583.26 | 1.60 | 0.080 | 0.018 |
| ~ bait + guide + trip density + proportion caught + muskellunge density + air temperature + barometric pressure + diel period + lunar position + solar radiation + wind direction | 15 | -1276.7 | 2583.67 | 2.00 | 0.065 | 0.023 |
| ~ bait + guide + trip density + proportion caught + muskellunge density + air temperature + barometric pressure + diel period + lunar position + solar radiation + wind direction + wind speed + wind speed:wind direction | 17 | -1275.2 | 2584.67 | 3.00 | 0.039 | 0.024 |
| ~ bait + guide + lunar position + solar radiation | 7 | -1285.5 | 2585.08 | 3.41 | 0.032 | 0.017 |
| ~ bait + guide + trip density + proportion caught + muskellunge density + air temperature + barometric pressure + diel period + lunar position + precipitation + solar radiation + wind direction + wind speed + wind speed:wind direction | 18 | -1275.2 | 2586.65 | 4.98 | 0.015 | 0.024 |
| ~ bait + guide + lunar position | 6 | -1287.7 | 2587.36 | 5.70 | 0.010 | 0.015 |
| ~ bait + lunar position | 5 | -1291.3 | 2592.67 | 11.00 | 0.001 | 0.013 |
| ~ bait + guide + trip density + proportion caught + muskellunge density + air temperature + barometric pressure + diel period + precipitation + solar radiation + wind direction + wind speed + wind speed:wind direction | 16 | -1281.9 | 2595.97 | 14.30 | 0.000 | 0.020 |
| ~ bait + guide + trip density + proportion caught + muskellunge density + air temperature + diel period + lunar phase + lunar position | 20 | -1277.9 | 2596.19 | 14.52 | 0.000 | 0.022 |
| ~ bait + guide + trip density + proportion caught + muskellunge density + air temperature + barometric pressure + diel period + lunar phase + lunar position + solar radiation + wind direction + wind speed + wind speed:wind direction | 24 | -1273.9 | 2596.27 | 14.60 | 0.000 | 0.025 |
| ~ bait + guide + trip density + proportion caught + muskellunge density + barometric pressure + diel period + lunar phase + lunar position + precipitation + solar radiation + wind direction + wind speed + wind speed:wind direction | 24 | -1274.3 | 2596.98 | 15.31 | 0.000 | 0.025 |
| ~ bait + guide + trip density + proportion caught + muskellunge density + air temperature + barometric pressure + diel period + lunar phase + lunar position + precipitation + solar radiation + wind direction | 23 | -1275.5 | 2597.38 | 15.71 | 0.000 | 0.024 |
| ~ bait + guide + trip density + proportion caught + muskellunge density + air temperature + diel period + lunar phase + lunar position + precipitation + solar radiation + wind direction + wind speed + wind speed:wind direction | 24 | -1274.6 | 2597.58 | 15.91 | 0.000 | 0.025 |
| ~ bait + guide + trip density + proportion caught + air temperature + barometric pressure + diel period + lunar phase + lunar position + precipitation + solar radiation + wind direction + wind speed + wind speed:wind direction | 24 | -1274.9 | 2598.18 | 16.51 | 0.000 | 0.024 |
| ~ bait + guide + trip density + proportion caught + muskellunge density + air temperature + barometric pressure + diel period + lunar phase + lunar position + precipitation + solar radiation + wind direction + wind speed + wind speed:wind direction | 25 | -1273.9 | 2598.28 | 16.61 | 0.000 | 0.025 |
| ~ bait + guide + proportion caught + muskellunge density + air temperature + barometric pressure + diel period + lunar phase + lunar position + precipitation + solar radiation + wind direction + wind speed + wind speed:wind direction | 24 | -1275.0 | 2598.48 | 16.81 | 0.000 | 0.024 |
| ~ bait + guide + trip density + muskellunge density + air temperature + barometric pressure + diel period + lunar phase + lunar position + precipitation + solar radiation + wind direction + wind speed + wind speed:wind direction | 24 | -1275.1 | 2598.63 | 16.96 | 0.000 | 0.024 |
| ~ bait + guide + trip density + proportion caught + muskellunge density + air temperature + barometric pressure + diel period + lunar phase + lunar position + precipitation + solar radiation + wind direction + wind speed | 24 | -1275.4 | 2599.23 | 17.56 | 0.000 | 0.024 |
| ~ bait + guide + trip density + air temperature + barometric pressure + diel period + lunar phase + lunar position + precipitation + solar radiation + wind direction + wind speed + wind speed:wind direction | 23 | -1276.6 | 2599.66 | 18.00 | 0.000 | 0.023 |
| ~ bait + guide + trip density + proportion caught + muskellunge density + air temperature + barometric pressure + lunar phase + precipitation + solar radiation + wind speed | 21 | -1278.7 | 2599.79 | 18.12 | 0.000 | 0.022 |
| ~ bait + guide + trip density + proportion caught + muskellunge density + air temperature + barometric pressure + diel period + lunar phase + lunar position + precipitation + wind direction + wind speed + wind speed:wind direction | 24 | -1275.9 | 2600.15 | 18.48 | 0.000 | 0.024 |
| ~ bait + guide + trip density + proportion caught + muskellunge density + air temperature + barometric pressure + lunar phase + lunar position + precipitation + wind direction + wind speed + wind speed:wind direction | 22 | -1277.9 | 2600.18 | 18.51 | 0.000 | 0.022 |
| ~ bait | 3 | -1297.9 | 2601.77 | 20.10 | 0.000 | 0.008 |
| ~ bait + trip density + proportion caught + muskellunge density + air temperature + barometric pressure + diel period + lunar phase + lunar position + precipitation + solar radiation + wind direction + wind speed + wind speed:wind direction | 24 | -1277.0 | 2602.37 | 20.70 | 0.000 | 0.023 |
| ~ guide + trip density + proportion caught + muskellunge density + air temperature + barometric pressure + diel period + lunar phase + lunar position + precipitation + solar radiation + wind direction + wind speed + wind speed:wind direction | 24 | -1278.0 | 2604.40 | 22.73 | 0.000 | 0.022 |
| ~ bait + guide + trip density + proportion caught + muskellunge density + air temperature + barometric pressure + diel period + lunar phase + precipitation + solar radiation + wind direction + wind speed + wind speed:wind direction | 23 | -1280.6 | 2607.55 | 25.88 | 0.000 | 0.020 |
| ~ trip density + proportion caught + muskellunge density + air temperature + barometric pressure + diel period + lunar phase + lunar position + precipitation + solar radiation + wind direction + wind speed + wind speed:wind direction | 23 | -1282.5 | 2611.37 | 29.70 | 0.000 | 0.019 |
| ~ proportion caught + muskellunge density + air temperature + barometric pressure + diel period + lunar phase + lunar position + precipitation + solar radiation + wind direction + wind speed + wind speed:wind direction | 22 | -1284.6 | 2613.63 | 31.96 | 0.000 | 0.018 |
| ~ null | 2 | -1309.2 | 2622.47 | 40.80 | 0.000 | 0.000 |


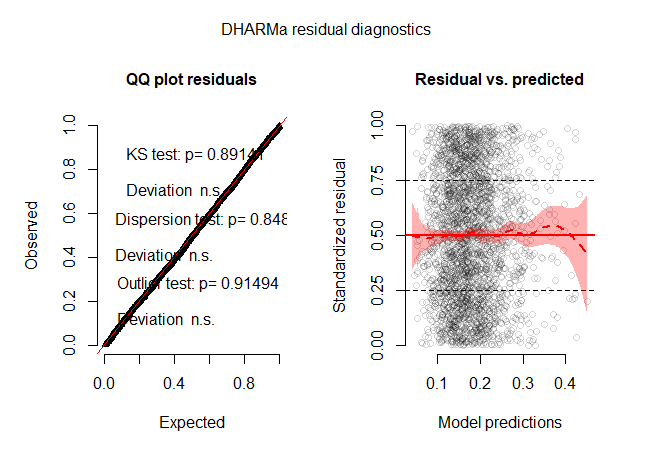


Fig 5. QQ-plot and residual versus predicted plot for the top ranked model exploring factors that affect muskellunge trip success on Escanaba Lake, WI, USA.

IV) *Muskellunge CPUE AICc model selection table, top model fit and variable effect sizes.*

Table 4. AICc model selection results for muskellunge *Esox masquinongy* positive truncated catch per unit effort data including model variables, model degrees of freedom, log likelihood (LL), AICc value, ΔAICc, model weight ($w_{t}$), model rank and log likelihood based R^2^ value (i.e., pseudo R^2^). Model variables included: bait type used (i.e., live or artificial; bait), whether or not a trip was guided (guide), daily trip density (i.e., boats per ha; trip density), the cumulative proportion of the walleye population caught and released (proportion caught), adult muskellunge density (i.e., number of muskellunge ≥ 30 inches /ha; walleye density), daily barometric pressure trend (barometric pressure), cumulative precipitation during trip (precipitation), mean daily solar radiation (solar radiation), daily mean wind speed (wind speed), direction of peak wind (wind direction), wind speed and direction interaction term (wind speed:wind direction), diel period (i.e., dawn, day or dusk; diel period), lunar phase (i.e., 8 factor characterization of moon phase; lunar phase), lunar position (i.e., overhead, underfoot or neither; lunar position), and daily mean air temperature (air temperature). If an interaction term is specified in the table then three terms were included in the model, one for each variable and one for the variable interaction. In all models including the null model year was included as a random effect (1|year).

| **Fixed Effects** | **df** | **LL** | **AIC_c_** | **ΔAIC_c_** | **w_t_** | **R^2^** |
| --- | --- | --- | --- | --- | --- | --- |
| ~ bait + trip density + proportion caught + air temperature + diel period + lunar phase + lunar position + wind speed | 19 | -436.56 | 912.69 | 0.00 | 0.220 | 0.177 |
| ~ bait + proportion caught + air temperature + diel period + lunar phase + lunar position + wind speed | 18 | -437.72 | 912.85 | 0.16 | 0.203 | 0.173 |
| ~ bait + trip density + proportion caught + air temperature + diel period + lunar phase + lunar position + wind direction + wind speed | 20 | -435.81 | 913.35 | 0.65 | 0.159 | 0.179 |
| ~ bait + air temperature + diel period + lunar phase + lunar position + wind speed | 17 | -439.12 | 913.51 | 0.81 | 0.147 | 0.168 |
| ~ bait + trip density + proportion caught + air temperature + diel period + lunar phase + lunar position + precipitation + wind direction + wind speed | 21 | -435.26 | 914.44 | 1.75 | 0.092 | 0.181 |
| ~ bait + trip density + proportion caught + air temperature + diel period + lunar phase + lunar position + precipitation + solar radiation | 22 | -434.73 | 915.56 | 2.87 | 0.052 | 0.183 |
| ~ bait + diel period + lunar phase + lunar position + air temperature | 16 | -441.53 | 916.17 | 3.48 | 0.039 | 0.160 |
| ~ bait + trip density + proportion caught + air temperature + diel period + lunar phase + lunar position + precipitation + solar radiation + wind direction + wind speed + wind speed:wind direction | 23 | -434.50 | 917.29 | 4.60 | 0.022 | 0.183 |
| ~ bait + diel period + lunar position | 8 | -450.78 | 917.84 | 5.15 | 0.017 | 0.129 |
| ~ bait + diel period + lunar phase + lunar position | 15 | -443.91 | 918.80 | 6.11 | 0.010 | 0.152 |
| ~ bait + trip density + proportion caught + air temperature + barometric pressure + diel period + lunar phase + lunar position + precipitation + solar radiation + wind direction + wind speed + wind speed:wind direction | 24 | -434.44 | 919.38 | 6.68 | 0.008 | 0.184 |
| ~ bait + guide + trip density + proportion caught + muskellunge density + air temperature + diel period + lunar phase + lunar position + solar radiation | 21 | -438.40 | 920.71 | 8.02 | 0.004 | 0.171 |
| ~ bait + guide + trip density + air temperature + barometric pressure + diel period + lunar phase + lunar position + precipitation + solar radiation + wind direction + wind speed + wind speed:wind direction | 24 | -435.13 | 920.76 | 8.07 | 0.004 | 0.181 |
| ~ bait + trip density + proportion caught + muskellunge density + air temperature + barometric pressure + diel period + lunar phase + lunar position + precipitation + solar radiation + wind direction + wind speed + wind speed:wind direction | 25 | -434.40 | 921.51 | 8.81 | 0.003 | 0.184 |
| ~ bait + guide + trip density + proportion caught + air temperature + barometric pressure + diel period + lunar phase + lunar position + precipitation + solar radiation + wind direction + wind speed + wind speed:wind direction | 25 | -434.43 | 921.57 | 8.87 | 0.003 | 0.184 |
| ~ bait + guide + trip density + proportion caught + muskellunge density + air temperature + barometric pressure + diel period + lunar phase + lunar position + precipitation + solar radiation + wind speed | 24 | -435.54 | 921.57 | 8.88 | 0.003 | 0.180 |
| ~ bait + guide + trip density + proportion caught + muskellunge density + air temperature + diel period + lunar phase + lunar position + precipitation + solar radiation + wind direction + wind speed + wind speed:wind direction | 25 | -434.44 | 921.59 | 8.90 | 0.003 | 0.184 |
| ~ bait + guide + trip density + proportion caught + muskellunge density + air temperature + barometric pressure + diel period + lunar phase + lunar position + precipitation + solar radiation + wind direction + wind speed | 25 | -434.61 | 921.94 | 9.24 | 0.002 | 0.183 |
| ~ proportion caught + muskellunge density + air temperature + barometric pressure + diel period + lunar phase + lunar position + precipitation + solar radiation + wind direction + wind speed + wind speed:wind direction | 23 | -437.06 | 922.42 | 9.73 | 0.002 | 0.175 |
| ~ trip density + proportion caught + muskellunge density + air temperature + barometric pressure + diel period + lunar phase + lunar position + precipitation + solar radiation + wind direction + wind speed + wind speed:wind direction | 24 | -435.99 | 922.47 | 9.78 | 0.002 | 0.179 |
| ~ bait + guide + trip density + proportion caught + muskellunge density + air temperature + barometric pressure + diel period + lunar phase + lunar position + precipitation + wind direction + wind speed + wind speed:wind direction | 25 | -434.91 | 922.54 | 9.84 | 0.002 | 0.182 |
| ~ bait + guide + trip density + proportion caught + muskellunge density + air temperature + barometric pressure + diel period + lunar phase + lunar position + solar radiation + wind direction + wind speed + wind speed:wind direction | 25 | -435.02 | 922.76 | 10.06 | 0.001 | 0.182 |
| ~ bait + guide + trip density + muskellunge density + air temperature + barometric pressure + diel period + lunar phase + lunar position + precipitation + solar radiation + wind direction + wind speed + wind speed:wind direction | 25 | -435.12 | 922.96 | 10.26 | 0.001 | 0.181 |
| ~ bait + guide + proportion caught + muskellunge density + air temperature + barometric pressure + diel period + lunar phase + lunar position + precipitation + solar radiation + wind direction + wind speed + wind speed:wind direction | 25 | -435.37 | 923.46 | 10.76 | 0.001 | 0.181 |
| ~ bait + guide + trip density + proportion caught + muskellunge density + air temperature + barometric pressure + diel period + lunar phase + lunar position + precipitation + solar radiation + wind direction + wind direction + wind speed + wind speed:wind direction | 26 | -434.38 | 923.69 | 11.00 | 0.001 | 0.184 |
| ~ bait + guide + trip density + proportion caught + muskellunge density + air temperature + barometric pressure + diel period + lunar phase + lunar position + precipitation + solar radiation + wind direction | 24 | -436.85 | 924.20 | 11.51 | 0.001 | 0.176 |
| ~ guide + trip density + proportion caught + muskellunge density + air temperature + barometric pressure + diel period + lunar phase + lunar position + precipitation + solar radiation + wind direction + wind speed + wind speed:wind direction | 25 | -435.83 | 924.38 | 11.68 | 0.001 | 0.179 |
| ~ bait + guide + trip density + proportion caught + muskellunge density + air temperature + barometric pressure + diel period + lunar position + precipitation + solar radiation + wind direction + wind speed + wind speed:wind direction | 19 | -442.95 | 925.48 | 12.78 | 0.000 | 0.156 |
| ~ bait + guide + trip density + proportion caught + muskellunge density + air temperature + barometric pressure + lunar phase + lunar position + precipitation + wind direction + wind speed + wind speed:wind direction | 23 | -441.00 | 930.29 | 17.60 | 0.000 | 0.162 |
| ~ bait + lunar position | 6 | -459.65 | 931.48 | 18.78 | 0.000 | 0.098 |
| ~ bait + guide + trip density + proportion caught + muskellunge density + air temperature + barometric pressure + lunar phase + lunar position + precipitation + solar radiation + wind direction + wind speed + wind speed:wind direction | 24 | -440.75 | 931.99 | 19.30 | 0.000 | 0.163 |
| ~ bait + guide + trip density + proportion caught + muskellunge density + air temperature + barometric pressure + diel period + precipitation + solar radiation + wind direction + wind speed + wind speed:wind direction | 17 | -453.80 | 942.85 | 30.16 | 0.000 | 0.119 |
| ~ bait + guide + trip density + proportion caught + muskellunge density + air temperature + barometric pressure + diel period + lunar phase + precipitation + solar radiation + wind direction + wind speed + wind speed:wind direction | 24 | -447.75 | 946.00 | 33.30 | 0.000 | 0.139 |
| ~ bait | 4 | -472.47 | 953.01 | 40.32 | 0.000 | 0.051 |
| ~ null | 3 | -485.68 | 977.42 | 64.72 | 0.000 | 0.000 |


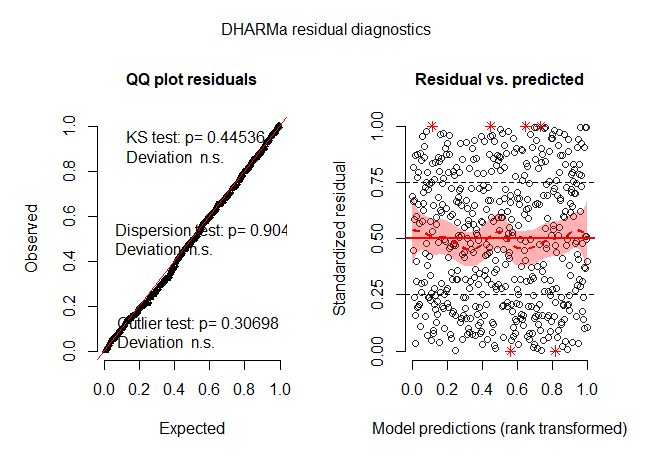


Fig 7. QQ-plot and residual versus predicted plot for the top ranked model exploring factors that affect muskellunge positive truncated catch per unit effort data on Escanaba Lake, WI, USA.
